# Automated deep learning segmentation of high-resolution 7 Tesla postmortem MRI for quantitative analysis of structure-pathology correlations in neurodegenerative diseases

**DOI:** 10.1162/imag_a_00171

**Published:** 2024-05-08

**Authors:** Pulkit Khandelwal, Michael Tran Duong, Shokufeh Sadaghiani, Sydney Lim, Amanda E. Denning, Eunice Chung, Sadhana Ravikumar, Sanaz Arezoumandan, Claire Peterson, Madigan Bedard, Noah Capp, Ranjit Ittyerah, Elyse Migdal, Grace Choi, Emily Kopp, Bridget Loja, Eusha Hasan, Jiacheng Li, Alejandra Bahena, Karthik Prabhakaran, Gabor Mizsei, Marianna Gabrielyan, Theresa Schuck, Winifred Trotman, John Robinson, Daniel T. Ohm, Edward B. Lee, John Q. Trojanowski, Corey McMillan, Murray Grossman, David J. Irwin, John A. Detre, M. Dylan Tisdall, Sandhitsu R. Das, Laura E. M. Wisse, David A. Wolk, Paul A. Yushkevich

**Affiliations:** Department of Bioengineering, University of Pennsylvania, Philadelphia, PA, United States; Penn Image Computing and Science Laboratory, University of Pennsylvania, Philadelphia, PA, United States; Department of Neurology, University of Pennsylvania, Philadelphia, PA, United States; Department of Radiology, University of Pennsylvania, Philadelphia, PA, United States; Department of Pathology and Laboratory Medicine, University of Pennsylvania, Philadelphia, PA, United States; Department of Diagnostic Radiology, Lund University, Lund, Sweden

**Keywords:** 7 T postmortem MRI, Alzheimer’s disease, dementia, deep learning, image segmentation

## Abstract

***Postmortem***MRI allows brain anatomy to be examinedat high resolution and to link pathology measures with morphometricmeasurements. However, automated segmentation methods for brain mapping inpostmortem MRI are not well developed, primarily due to limited availability oflabeled datasets, and heterogeneity in scanner hardware and acquisitionprotocols. In this work, we present a high-resolution dataset of 135 postmortemhuman brain tissue specimens imaged at 0.3 mm^3^isotropic using a T2wsequence on a 7T whole-body MRI scanner. We developed a deep learning pipelineto segment the cortical mantle by benchmarking the performance of nine deepneural architectures, followed by post-hoc topological correction. We evaluatethe reliability of this pipeline via overlap metrics with manual segmentation in6 specimens, and intra-class correlation between cortical thickness measuresextracted from the automatic segmentation and expert-generated referencemeasures in 36 specimens. We also segment four subcortical structures (caudate,putamen, globus pallidus, and thalamus), white matter hyperintensities, and thenormal appearing white matter, providing a limited evaluation of accuracy. Weshow generalizing capabilities across whole-brain hemispheres in differentspecimens, and also on unseen images acquired at 0.28 mm^3^and 0.16mm^3^isotropic T2*w fast low angle shot (FLASH) sequence at7T. We report associations between localized cortical thickness and volumetricmeasurements across key regions, and semi-quantitative neuropathological ratingsin a subset of 82 individuals with Alzheimer’s disease (AD) continuumdiagnoses. Our code, Jupyter notebooks, and the containerized executables arepublicly available at the**project webpage**(https://pulkit-khandelwal.github.io/exvivo-brain-upenn/).

## 1 Introduction

Neurodegenerative diseases are increasingly understood to be heterogeneous, withmultiple distinct neuropathological processes jointly contributing toneurodegeneration in most patients, called*mixed pathology*(Schneider et al., 2007). For example, manypatients diagnosed at autopsy with Alzheimer’s disease (AD) also have brainlesions associated with vascular disease, TDP-43 proteinopathy, andα-synuclein pathology (Matej et al.,2019;Robinson et al., 2018).Currently, some of these pathological processes (particularly TDP-43 andα-synuclein pathologies) cannot be reliably detected with antemortembiomarkers, which makes it difficult for clinicians to determine to what extentcognitive decline in individual patients is driven by AD versus other factors. Therecent modest successes of AD treatments in clinical trials (van Dyck et al., 2022) make it ever more important toderive antemortem biomarkers that can detect and quantify mixed pathology, so thattreatments can be prioritized for those most likely to benefit from them.

Importantly, the understanding and utility of antemortem biomarkers are augmented bythe coupling of imaging and postmortem pathology. Following autopsy, histologicalexamination of the donor’s brain tissue provides a semi-quantitativeassessment of the presence and severity of various pathological drivers ofneurodegeneration. Associations between these pathology measures and regionalmeasures of neurodegeneration, such as cortical thickness, can identify patterns ofneurodegeneration probabilistically linked to specific pathological drivers (de Flores et al., 2020;Frigerio et al., 2021;Wisse et al., 2020,^2021^). Suchassociation studies can use either antemortem or postmortem imaging (Wisse et al., 2017). Both approaches have theirlimitations. When the time between antemortem imaging and death is substantial, thepostmortem pathology may not accurately match the state of pathology at the time ofimaging. Postmortem MRI generally requires dedicated imaging facilities and imageanalysis algorithms, and whatever neurodegeneration/pathology association patternsare discovered have to be “translated” into the antemortem imagingdomain for use as antemortem biomarkers. However, postmortem MRI allows imaging atmuch greater resolution than antemortem, allowing structure/pathology associationsto be examined with greater granularity than antemortem imaging.

Furthermore, postmortem MRI of the brain can provide an advantage over antemortem MRIfor visualizing detailed and intricate neuroanatomy and linking macroscopicmorphometric measures such as cortical thickness to underlying cytoarchitecture andpathology (Adler et al., 2014;Alkemade et al., 2022;Augustinack et al., 2010;Beaujoin etal., 2018;García-Cabezas et al.,2020;Iglesias et al., 2015;Mancini et al., 2020;Pallebage-Gamarallage et al., 2018;Vega et al., 2021). Recent inquiries comparing postmortemimaging with histopathology have demonstrated relationships between atrophy measuresand neurodegenerative pathology (Makkinejad et al.,2019;Ravikumar et al., 2021;Wisse et al., 2021;Yushkevich et al., 2021). Such associations corroboratepatterns of neurodegeneration by specifically linking them with the underlyingcontributing pathology such as TAR DNA-binding protein 43 (TDP-43), phosphorylatedtau (p-tau), and α-synuclein in Alzheimer’s Disease (AD). Inparticular,Ravikumar et al. (2021)foundsignificant correlations between tau pathology and thickness in the entorhinalcortex (ERC) and stratum radiatum lacunosum moleculare (SRLM) consistent with earlyBraak stages. Separately,Wisse et al. (2021)found significant associations of TDP-43 with thickness in the hippocampalsubregions. Postmortem imaging also helps in characterizing underlying anatomy atthe scale of subcortical layers (Augustinack et al.,2013;Kenkhuis et al., 2019), suchas hippocampal subfields in the medial temporal lobe (MTL) (Ravikumar et al., 2020;Yushkevich et al., 2021). Several studies have also exploredpathology/MRI associations in other neurodegenerative diseases, such asfrontotemporal lobar degeneration (FTLD) and amyotrophic lateral sclerosis (ALS)(Gordon et al., 2016;Irwin et al., 2015;Mackenzie et al., 2011). Previous work has identified correlationsbetween high-resolution postmortem MRI and histopathology, to map myelin and irondeposits in cortical laminae (Bulk et al.,2020), due to oligodendrocytes and pathologic iron inclusions inastrocytes and microglia (Tisdall et al.,2021), which are a major source of iron because of myelination demands.Therefore, postmortem MRI would be helpful for validating and refiningpathophysiological correlates derived from antemortem studies. Additionally, thevolume of the WMH burden is an indirect marker of cerebrovascular pathology and theassociations between cortical thickness and WMH (Dadar et al., 2022;Du et al.,2005;Rizvi et al., 2018) arecomplementary to the associations between cortical thickness and tau, TDP-43,amyloid-βand α-synuclein pathology. Also,van derVelpen et al. (2023)suggest that the subcortical brain structures arehighly involved in dementia risk. Smaller volumes and thickness measurements ofthalamus, amygdala, and hippocampus were associated with incident dementia.

Compared to antemortem MRI, postmortem MRI is not affected by head or respiratorymotion artifacts and has much less stringent time. Compared to histology, postmortemMRI is less affected by distortion or tearing, and provides a continuous 3Drepresentation of brain anatomy. However, both histology and postmortem MRI areaffected by changes occurring in the agonal state, during brain removal, and duringtissue fixation and handling. Indeed, postmortem MRI is often used to provide a 3Dreference space onto which 2D histological images are mapped. Combined analysis ofpostmortem MRI and histology makes it possible to link morphological changes in thebrain to underlying pathology as well as to generate anatomically correctparcellations of the brain based on cytoarchitecture (Amunts et al., 2020;Schiffer, Spitzer,et al., 2021), and pathoarchitecture (Augustinack et al., 2013). Postmortem MRI could also act as a referencespace to generate quantitative 3D maps of neurodegenerative proteinopathies fromserial histology imaging (Ushizima et al.,2022;Yushkevich et al.,2021).

Given the rising use of high-resolution postmortem MRI in neurodegenerative diseaseresearch, automated techniques are imperative to effectively analyze such growingdatasets. Particularly, in the case of structure-pathology association studies,scaling them beyond a few dozen datasets requires reliable morphometry measurementsfrom postmortem MRI via accurate 3D segmentation and reconstruction of thestructures of interest. There has been substantial work in brain MRI parcellationsuch as*FreeSurfer*(Fischl,2012) and recent efforts based on deep learning (Chen et al., 2018;Henschel et al., 2020). However, these approaches focus on antemortemMRI, and there is limited work on developing automated segmentation methods forpostmortem MRI segmentation. Postmortem segmentation methods have been regionspecific. Recent developments include automated deep learning methods forhigh-resolution cytoarchitectonic mapping of the occipital lobe in 2D histologicalsections (Eckermann et al., 2021;Kiwitz et al., 2020;Schiffer, Amunts, et al., 2021;Schiffer, Harmeling, et al., 2021;Schiffer, Spitzer, et al., 2021;Spitzer et al., 2018). The work byIglesias et al. (2015,^2018^) has developed an atlas to segment the MTL and the thalamus usingmanual segmentations in postmortem images. Yet, a postmortem segmentation methodapplicable to a variety of brain regions has yet to be described. This isattributable to several factors. Some groups have developed robust whole-brainpostmortem image analysis tools (Casamitjana et al.,2024;Edlow et al., 2019;Jonkman et al., 2019;Mancini et al., 2020;Zeng et al., 2023), though overall there is limited availability ofpostmortem specimens, scans, segmentation algorithms, and labeled reference standardsegmentations. Compared to antemortem structural MRI, postmortem MRI currentlyexhibits substantial heterogeneity in scanning protocols, larger image dimensions,greater textual complexity, and more profound artifacts. These issues can beaddressed with new datasets and automated segmentation tools open to the public.

In this study, we expand upon our pilot study (Khandelwal, Duong, et al., 2022;Khandelwal, Sadaghiani, et al., 2022;Khandelwal et al., 2021) and develop a methodological framework tosegment cortical gray matter; subcortical structures (caudate, putamen, globuspallidus, thalamus), white matter (WM), and WMH in high-resolution (0.3 x 0.3 x 0.3mm^3^) 7 T T2w postmortem MRI scans of whole-brain hemispheres. Wetrain and evaluate our approach using 135 brain hemisphere scans from the Center forNeurodegenerative Disease Research of the University of Pennsylvania. We measurecortical thickness at several key locations in the cortex based on our automaticsegmentation of the cortex, and correlate these measures with thickness measurementsobtained using a user-guided semi-automated protocol. High consistency between thesetwo sets of measures supports the use of deep learning-based automated thicknessmeasures for postmortem brain segmentation and morphometry. We then report regionalpatterns of association between cortical thickness at a set of anatomical locationsand neuropathology ratings (regional measures of p-tau, neuronal loss; as well asglobalamyloid-β,Braak staging, and CERAD ratings) obtained from histology data and WMH burden.Additionally, we show that networks trained on T2-weighted spin echo images acquiredat 7 T generalize to postmortem images obtained with T2*w gradient echo fastlow angle shot (FLASH) 7T MRI acquired at a resolution of 0.28 x 0.28 x 0.28mm^3^and 0.16 x 0.16 x 0.16 mm^3^.

## 2 Materials

### 2.1 Donor cohort

**Fig. 1.**
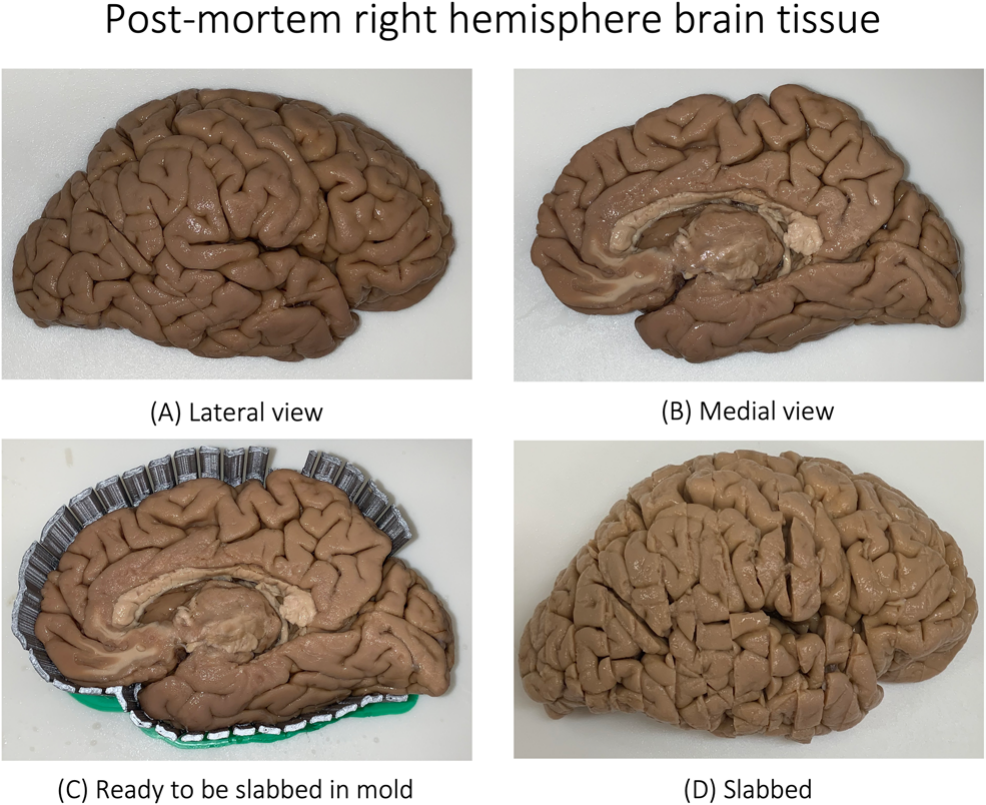
Postmortem tissue blockface photograph of a donor with diagnosis ofParkinson’s disease (not demented) and Lewy body disease(deceased at the age of 79). Shown are the lateral (A) and medial (B)views of the right hemisphere. The tissue is then placed in a mold (C)and is subsequently slabbed (cut into different slices) as shown (D).SeeLasserve et al., 2020formore details. SeeSupplementary Figure 1for the slices of the given braintissue.

**Fig. 2.**
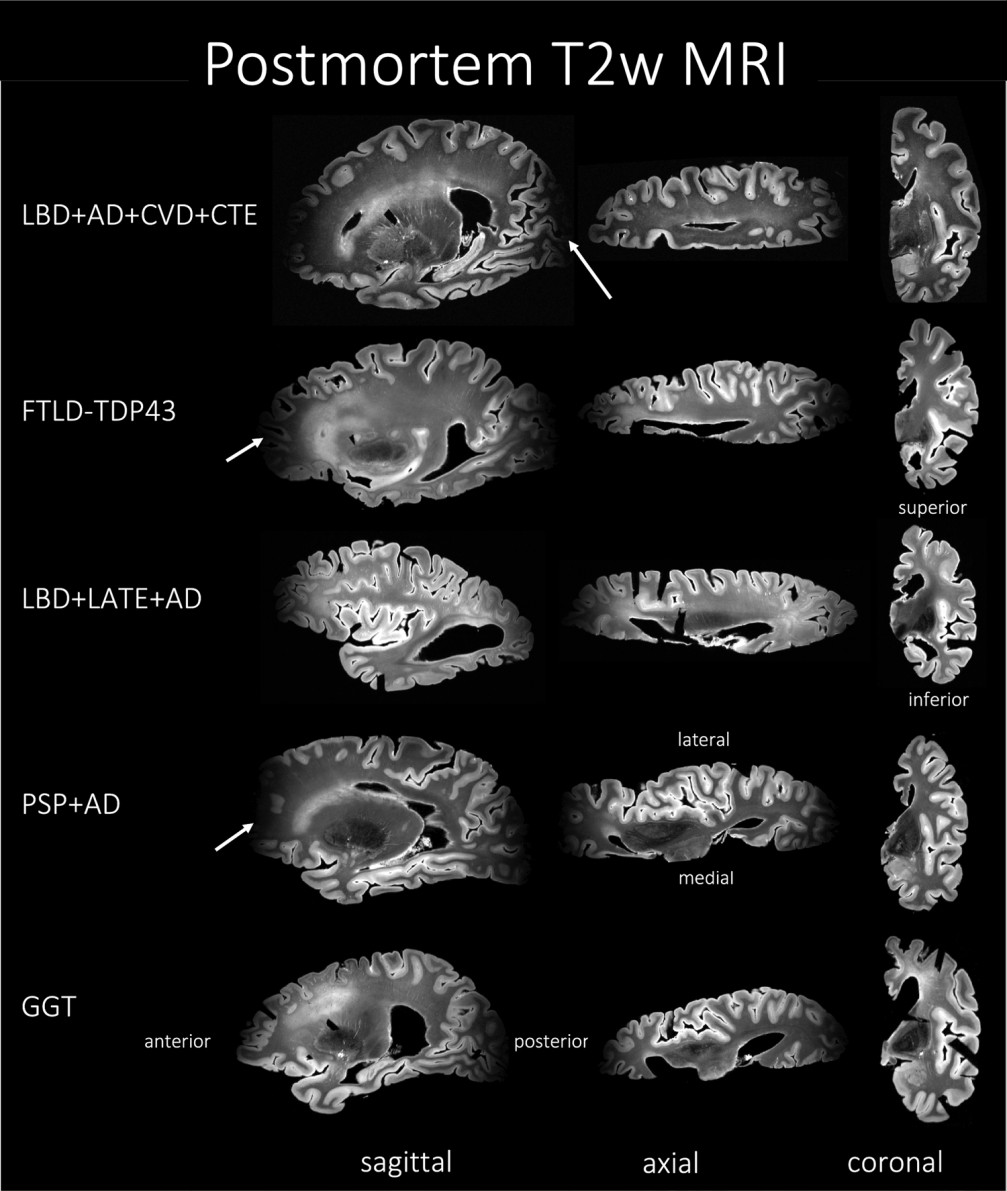
MRI of the T2w sequence representative of Alzheimer’s disease andrelated dementias (ADRD) spectrum with mixed pathology and diagnoses offive subjects. The heterogeneity among the subjects can be appreciatedthrough the three different viewing planes. Notice that the MRI signaldrops off at the anterior and posterior ends of the hemisphere, adrawback of the current acquisition protocol. AD, Alzheimer’sdisease; CVD, cerebrovascular disease; LATE, limbic-predominantage-related TDP-43 encephalopathy; LBD, Lewy body disease; CTE, chronictraumatic encephalopathy; FTLD-TDP, frontotemporal lobar degenerationwith TDP inclusions; GGT, globular glial tauopathy; PART, primaryage-related tauopathy; PSP, progressive supranuclear palsy.

We analyze a dataset of 135 postmortem whole-hemisphere MRI scans selected fromPenn Integrated Neurodegenerative Disease Database (INDD) (Toledo et al., 2014). Patients were evaluated at thePenn Frontotemporal Degeneration Center (FTDC) or Alzheimer’s DiseaseResearch Center (ADRC) and followed to autopsy at the Penn Center forNeurodegenerative Disease Research (CNDR) as part of ongoing and previousclinical research programs (Arezoumandan et al.,2022;Irwin, 2016;Tisdall et al., 2021). The cohort included62 female (sex assigned at birth) donors (Age: 75.37±10.02 years, Age range: 53-97) and 73 male donors (Age: 73.95±11.59 years, Age range: 44–101) with Alzheimer’s Disease orrelated dementias (ADRD), such as Lewy body disease (LBD), FTLD-TDP43,progressive supranuclear palsy (PSP), or cognitively normal adults. Human brainspecimens were obtained in accordance with local laws and regulations, andincluded informed consent from next of kin at time of death. The patients wereevaluated at FTDC and ADRC as per standard diagnostic criteria (Toledo et al., 2014), and imaged by the teams at theADRC and the Penn Image Computing and Science Laboratory (PICSL) and the FTDC.Autopsy was performed at the CNDR.Figure 1shows an example of a brain specimen with Parkinson’s and LBD ready forautopsy. The brain is subsequently cut into 1 cm-thick slabs using a 3D-printedcutting mold, and slabs are further cut and embedded in paraffin for otherongoing studies in the laboratory.Supplementary Figure 1illustrates the internalstructures of the brain. Separately, the postmortem tissue photograph of aspecimen with progressive non-fluent aphasia (PFNA) and Globular glial tauopathy(GGT) disease is shown inSupplementary Figure 2.Table1details the primary neuropathological diagnostic groups in thecohort with complete details tabulated in theSupplementarySpreadsheet.

**Table 1.** Demographics of the Alzheimer’s disease and related dementias(ADRD) patient cohort in the current study.

Brain donor cohort				
N	135 (Female: 62 and Male: 73)			
Age (years)	74.60 ± 10.88 (range 44-101)			
Race	White: 125 Black: 8 More than one race: 1 Unknown: 1			
Hemisphere imaged	Right: 66 Left: 69			
Postmortem interval (hours)	20.22 ± 15.05 (range: 3-105)			
Fixation time (days)	298.67 ± 338.60 (range: 29-1559)			

Neuropathological diagnosis				
	Primary	Secondary/tertiary		
Alzheimer’s disease	53	40		
Lewy body disease	25	26		
FTLD-TDP	17	-		
Pick’s disease	9	-		
Progressive supranuclear palsy	6	1		
Cerebrovascular disease	5	5		
Corticobasal degeneration	4	1		
Globular glial tauopathy	4	-		
PART	2	10		
LATE	2	15		
Amyotrophic lateral sclerosis	1	1		
FTLD-FUS	1	-		
FTD with Parkinsonism (chromosome 17)	1	-		
Argyrophilic grain disease	-	4		
Hippocampal Sclerosis	-	5		

Global neuropathological staging				
Ratings	0	1	2	3
Amyloid- β Thal staging (A score)	26	21	32	56
Braak three stage scheme (B score)	14	34	27	48
CERAD (C score)	49	20	16	50

AD, Alzheimer’s disease; ALS, Amyotrophic lateral sclerosis;CVD, Cerebrovascular disease; LATE, Limbic-predominant age-relatedTDP-43 encephalopathy; LBD, Lewy body disease; CTE, chronictraumatic encephalopathy; FTLD-TDP, Frontotemporal lobardegeneration with TDP inclusions; GGT, Globular glial tauopathy;CVD, Cerebrovascular disease; CBD, Corticobasal degeneration; PART,Primary age-related tauopathy; PSP, Progressive supranuclear palsy;FUS, Fused-in-Sarcoma; tau-Misc, tauopathy unclassifiable.

### 2.2 MRI acquisition

During the autopsy of a specimen, one hemisphere was immersed in10%neutral buffered formalin for at least 4 weeks prior to imaging. After thefixation time, samples were placed in Fomblin (California Vacuum Technology;Freemont, CA), enclosed in custom-built plastic bag holders. Samples were leftto rest for at least 2 days to allow the air bubbles to escape from the tissue.The samples were scanned using either a custom-built small solenoid coil or acustom-modified quadrature birdcage (Varian, Palo Alto, CA, USA) coil (Edlow et al., 2019;Tisdall et al., 2021). The samples were then placedinto a whole-body 7 T scanner (MAGNETOM Terra, Siemens Healthineers, Erlangen,Germany). T2-weighted images were acquired using a 3D-encoded T2 SPACE sequencewith 0.3 x 0.3 x 0.3 mm^3^isotropic resolution, 3 s repetition time(TR), echo time (TE) 383 ms, turbo factor 188, echo train duration 951 ms, andbandwidth 348 Hz/px in approximately 2–3 hours per measurement. Imagereconstruction was done using the vendor’s on-scanner reconstructionsoftware which also corrected the global frequency drift, combined the signalaverages in k-space, and produced magnitude images for each echo. A total offour repeated measurements were acquired for each sample and subsequentlyaveraged to generate the final image. Sample MRI slices are shown inFigure 2for a range of specimens withdifferent diseases. The image acquisition suffers from geometric distortions dueto the non-linearity of the magnetic gradient field that increases towards theends (both anterior and posterior) of the sample and B1-transmit inhomogeneity,which results in decreased image quality as shown inFigure 2.

We also acquire images at a much higher resolution using two separate T2*wFLASH MRI sequences, one at 280 microns and the other at 160 microns. We usethese sequences for the generalization experiments as described inSection 4.3. Here, we briefly describe theiracquisition parameters. For the 280 microns isotropic resolution: MRI data werereacquired with a 3D-encoded, 8-echo gradient-recalled echo (GRE) sequence withnon-selective RF pulses. To maintain readout polarity and minimize distortionsdue to field inhomogeneity, each readout was followed by a flyback rephrasinggradient. The final echo was followed by an additional completely rephrasedreadout to measure frequency drifts. Each line of k-space was acquired withmultiple averages sequentially before advancing to the next phase-encode step.Common parameters for the sequence were: 280 microns isotropic resolution,25°flip angle, 60 ms repetition time (TR), minimum echo time (TE) 3.48 ms, echospacing 6.62 ms, and bandwidth 400 Hz/px. The field of view was adapted to eachsample, and subsequently TRs and TEs were slightly modified based on thenecessary readout duration. Total scan times were 8–10 hours for eachsample.

For the 160 microns isotropic resolution: MRI data were acquired with a3D-encoded, 3-echo GRE sequence with non-selective RF pulses with the sequence:25°flip angle, 60 ms repetition time (TR), minimum echo time (TE), 9.37 ms, echospacing 11.33 ms, and bandwidth 90 Hz/px similar to methods described inTisdall et al. (2022).

### 2.3 Neuropathological ratings

The non-imaged hemisphere, that is, the contralateral tissue of each specimensystematically underwent histological processing for neuropathologicalexamination at the CNDR (Toledo et al.,2014). Roughly 1.5 × 1.5 × 0.5 cm^3^tissueblocks were extracted from the contralateral hemisphere. Paraffin-embeddedblocks were then sectioned into 6μmfor immunohistochemistry using phosphorylated tau (AT8; Fisher, Waltham, MA;Catalogue No. ENMN1020) to detect phosphorylated p-tau deposits and p409/410(mAb, 1:500, a gift from Dr. Manuela Neumann and Dr. E. Kremmer) to detectphosphorylated TDP-43 deposits. Immunohistochemistry evaluation was performed onthe hemisphere contralateral to the hemisphere that was scanned using previouslyvalidated antibodies and established methods: NAB228 (monoclonal antibody [mAb],1:8000, generated in the CNDR), phosphorylated tau PHF-1 (mAb, 1:1000, a giftfrom Dr. Peter Davies), TAR5P-1D3 (mAb, 1:500, a gift from Dr. Manuela Neumannand Dr. E. Kremmer), and Syn303 (mAb, 1:16,000, generated in the CNDR) to detectamyloidβ(Aβ)deposits, phosphorylated tau (p-tau) deposits, phosphorylated TDP-43 deposits,and the presence of pathological conformation of α-synuclein,respectively. In 16 cortical regions, semi-quantitative ratings of p-tau,TDP-43,β-amyloid,and α-synuclein pathology, as well as neuronal loss, were visuallyassigned by an expert neuropathologist (E.B.L. and J.Q.T.) on a scale of0–3, that is, “0: None,” “0.5: Rare,”“1: Mild,” “2: Moderate,” or “3:Severe” (Toledo et al., 2014).These ratings are illustrated inFigure 3.Global ratings of neurodegenerative disease progression were also derived,including A, B, and C scores (Hyman et al.,2012).Supplementary Table 2details the locations from where theneuropathology ratings were obtained from, either the exact (main regions) orthe closest (exploratory regions), to the cortical brain regions. Thejustification of this matching of neuropathology regions to regions on MRI isprovided inSadaghiani et al. (2022).

**Fig. 3.**
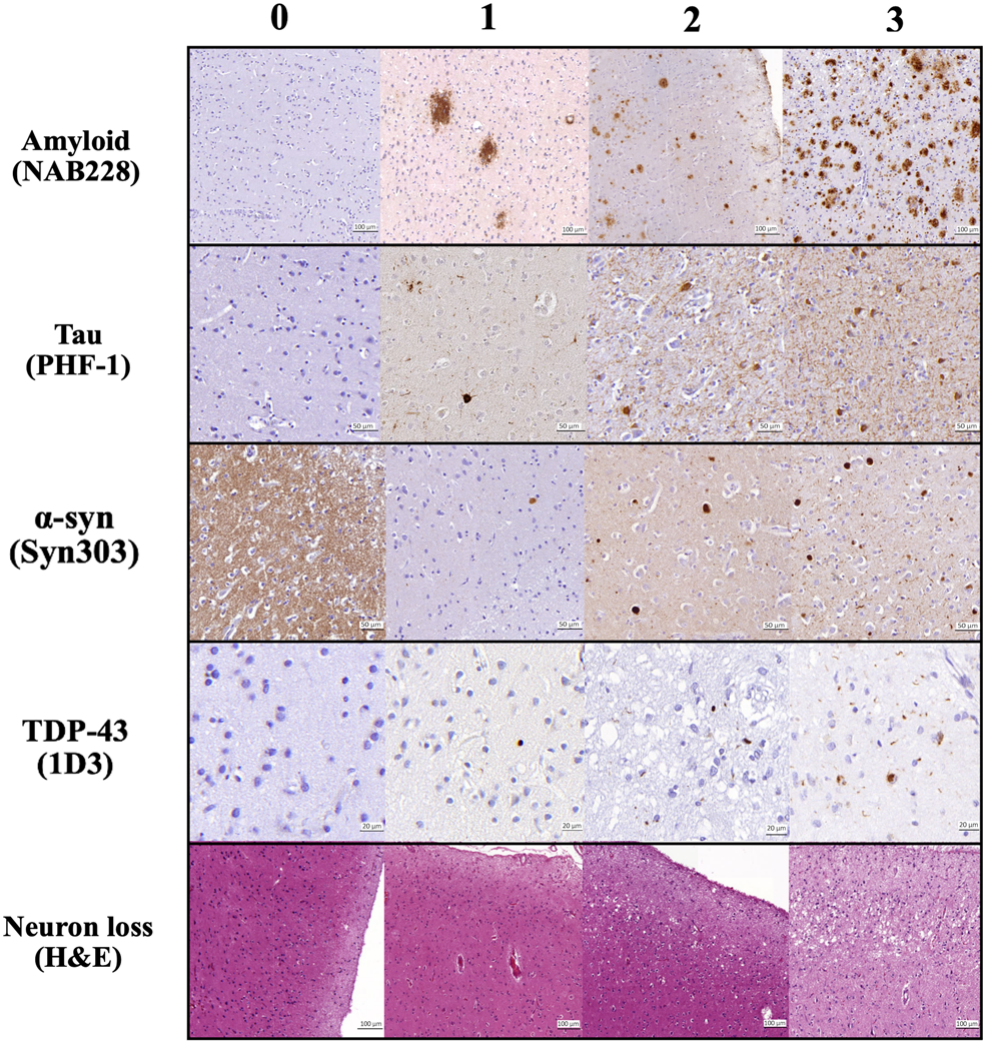
Pathological assessment for each regional pathology derived fromhistology as per the protocol discussed inSection 2.3. The columns are severity ratings (left toright): 0-3. The shown pathologies are: amyloid-βplaques (NAB228 antibody), p-tau pathology (PHF-1 antibody),α-synuclein (Syn303 antibody), TDP-43 inclusions (pS409/410antibody), and neuron loss (Hematoxylin and Eosin staining) in differentcortical and medial temporal lobe regions included in the currentwork.

### 2.4 Localized thickness measurement pipeline at key cortical locations

**Fig. 4.**
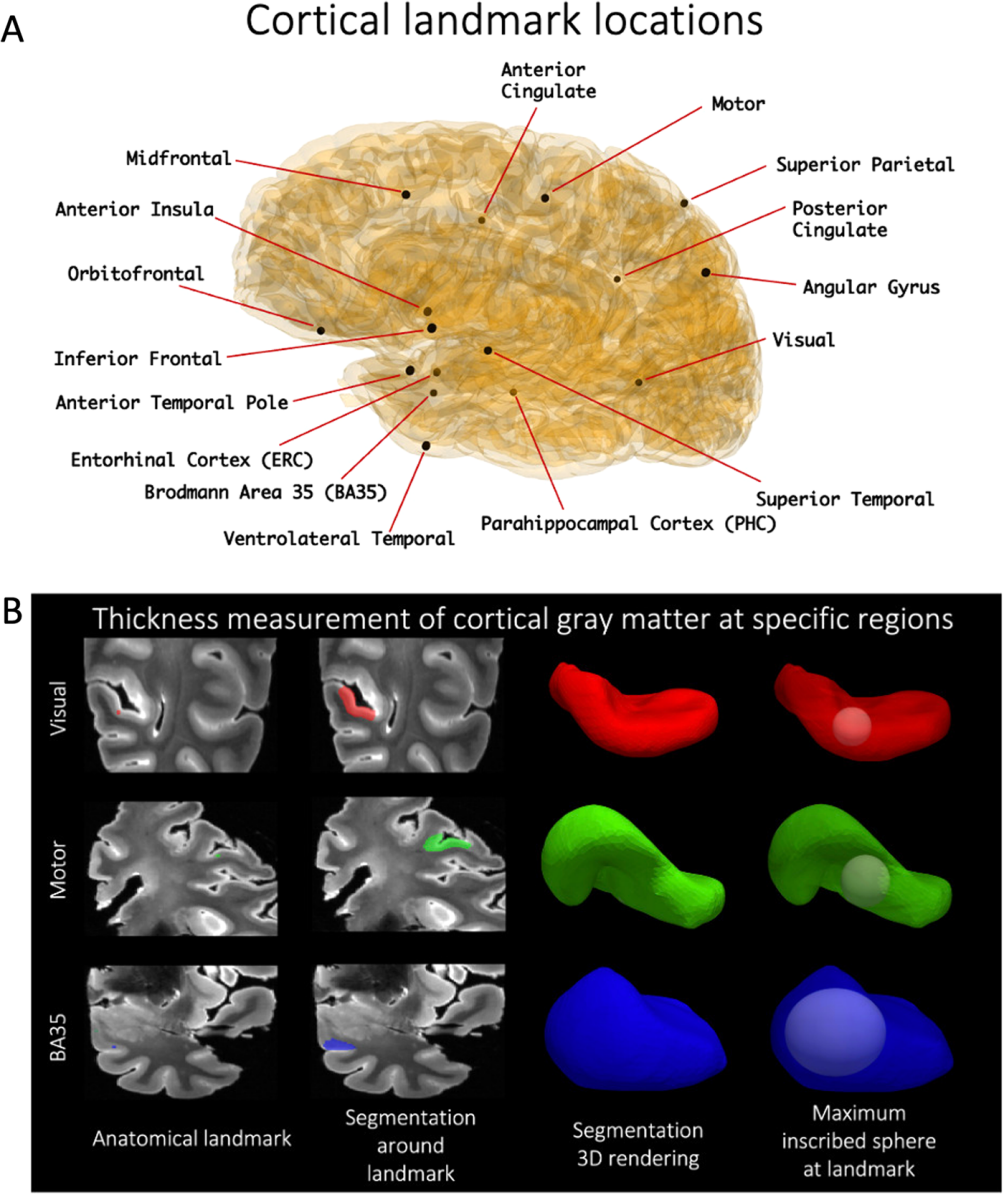
Cortical thickness is measured at the 16 landmarks (A). A dot (shownhere: motor, visual, BA35) is first placed to define an anatomicallandmark, around which a semi-automatic level set segmentation of thesurrounding cortical ribbon is provided. A maximally inscribed sphere isthen computed using Voronoi skeletonization (Ogniewicz & Ilg, 1992), and thediameter of the sphere gives thickness at that landmark (B).

Our center has adopted an expert-supervised semi-automatic protocol to obtainlocalized quantitative measures of cortical thickness in all 7 T postmortem MRIscans, as described in the work bySadaghiani etal. (2022)Section 2.3andsupplementary materialS1andWisse et al. (2021)Section: MTL thickness measurements andsupplementary material: Thickness measurement. In thecurrent study, we use these measures as the reference standard for evaluatingautomated cortical segmentation. In each hemisphere, 16 cortical landmarks areidentified and labeled on the MRI scan as shown inFigure 4A. To measure cortical thickness at these locations, asemi-automatic level set segmentation of the surrounding cortical ribbon isperformed and the maximal sphere inscribed into the cortical segmentation andcontaining the landmark is found; the diameter of this sphere gives thickness atthat landmark (Fig. 4B).

## 3 Methods

InSection 3.1, we describe the manualsegmentation protocols developed for cortical gray matter, the four subcorticalstructures, WMH, and normal-appearing white matter. Then, inSections 3.2and3.3, wedescribe the deep learning-based pipeline and performance evaluation criteriadeveloped for the automated segmentation of these structures. InSection 3.4, we describe the post-hoc topologycorrection step employed to provide geometrically accurate segmentations. Finally,inSection 3.5, we describe the statisticalmodels used to link neuropathological ratings of p-tau,amyloid-βneuronal loss, CERAD scores, Braak Staging with morphometry based on volume, andregional thickness measurements obtained from the automated segmentations.

### 3.1 Manual segmentation protocols

We developed protocols to manually segment structures in postmortem MRI: corticalgray matter, subcortical structures (caudate, putamen, globus pallidus,thalamus), WM, and WMH. We used these manual segmentations along with thecorresponding MR images to train the neural networks. All manual segmentationswere done by raters in ITK-SNAP (Yushkevich etal., 2019).Supplementary Table 1shows which subjects were manually segmentedto obtain the training data and the reference standard for the different labels,and the subsequent cross-validation studies as described inSections 3.2and3.3.

#### 3.1.1 Cortical gray matter

**Fig. 5.**
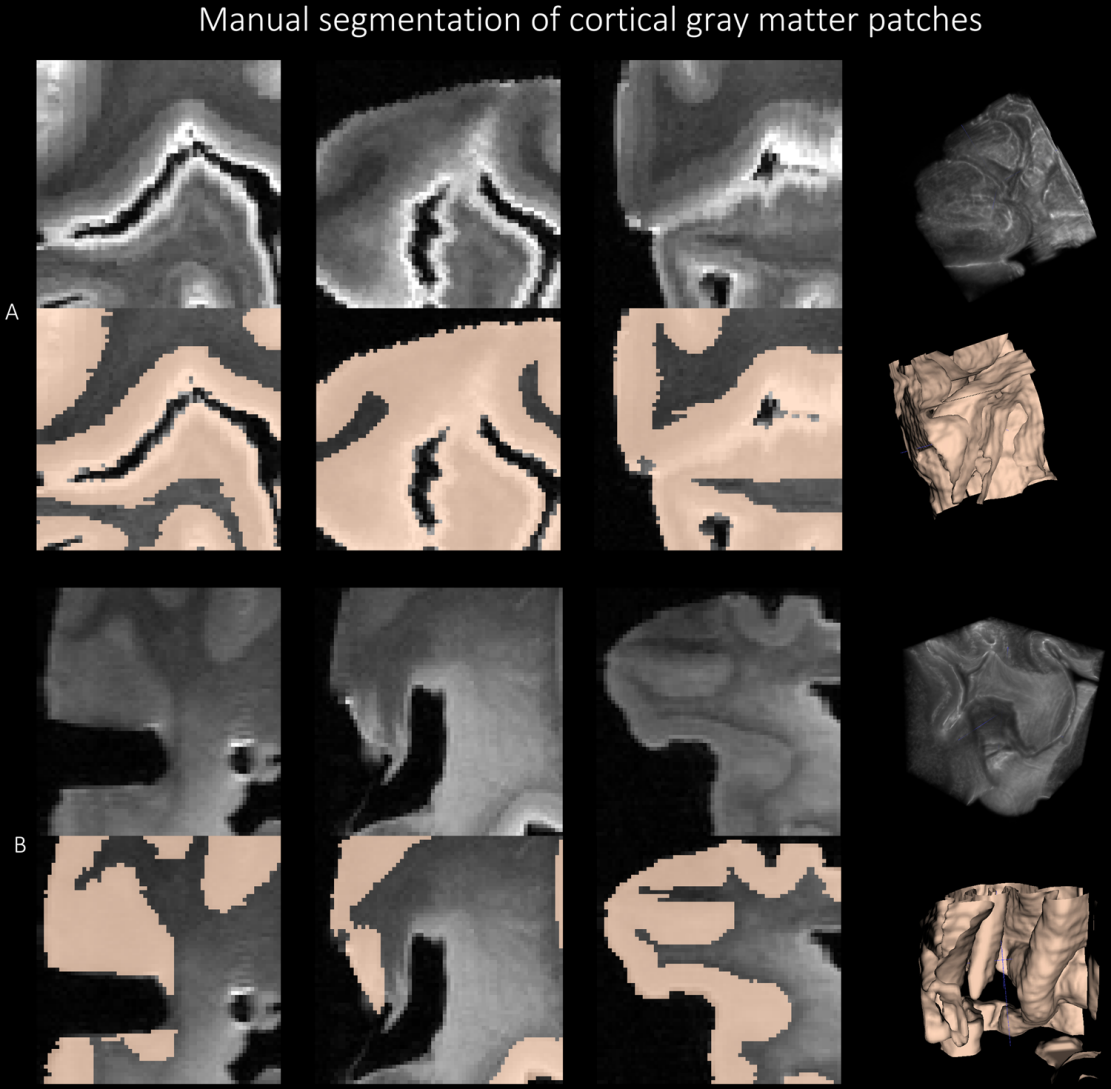
Manual segmentation of the cortical gray matter patches of size 64 x64 x 64 using the protocol explained inFigure 5.Section3.1.1for two subjects with FTLD-TDP (A) and GGT (B) inthree viewing planes with the corresponding 3D renderings.

We sampled five 3D image patches of size 64 x 64 x 64 voxel^3^, asshown inFigure 5around theorbitofrontal cortex (OFC), anterior temporal cortex (ATC), inferiorprefrontal cortex (IPFC), primary motor cortex (PMC), and primarysomatosensory cortex (PSC) from 6 brain hemispheres, resulting in a total of30 patches. These regions were selected as representative regions withvariable levels of pathology in ADRD cases and control regions (PMC, PSC)(Tisdall et al., 2021). Forexample, OFC, ATC, and IPFC have high pathology in PSP, whereas, the PSC,which is generally less affected in most neurodegenerative diseases, wassampled as a negative control. In each patch, gray matter was segmentedmanually in ITK-SNAP. Five manual raters, divided into groups of two (E.K.and G.C.) and three (E.H., B.L. and E.M.), labeled gray matter as theforeground, and rest of the image as the background using a combination ofmanual tracing and the semi-automated segmentation tool. The manualsegmentation of cortical gray matter was supervised by author P.K.Figure 5shows sample patch images andthe corresponding reference standard labels with 3D renderings. We followedsome guiding principles for manual segmentation: (1) we note that the whitelayer enveloping the cortex is not an imaging artifact, but is the outermostlayer of the cortex, and thus is labeled as the gray matter; (2) adjoininggyri in deep sulci region are correctly labeled as gray matter and demarcatethe deep sulci as the background; (3) in several regions, gray matter havesimilar intensities with the nearby WMH which were resolved by visualinspection of texture in the surrounding region; and (4) the 64 x 64 x 64patches provided little context when segmenting the GM, therefore, thecorresponding whole hemisphere image was displayed on a separate ITK-SNAPwindow for the user to examine the structures surrounding the given imagepatch. Inter-rater reliability scores were computed for these manualsegmentations in terms of Dice Coefficient (DSC): Raters 1&2: 95.26±1.37 %, Raters 1&3: 94.64±1.64 %, Raters 2&3: 94.54±1.20 %, and Raters 4&5: 92.04±4.26 %. After the review of the segmentations by an expert, the patchessegmented by raters 2 (G.C.) and 5 (E.M.) were selected for training thedeep learning models.

#### 3.1.2 Subcortical structures

**Fig. 6.**
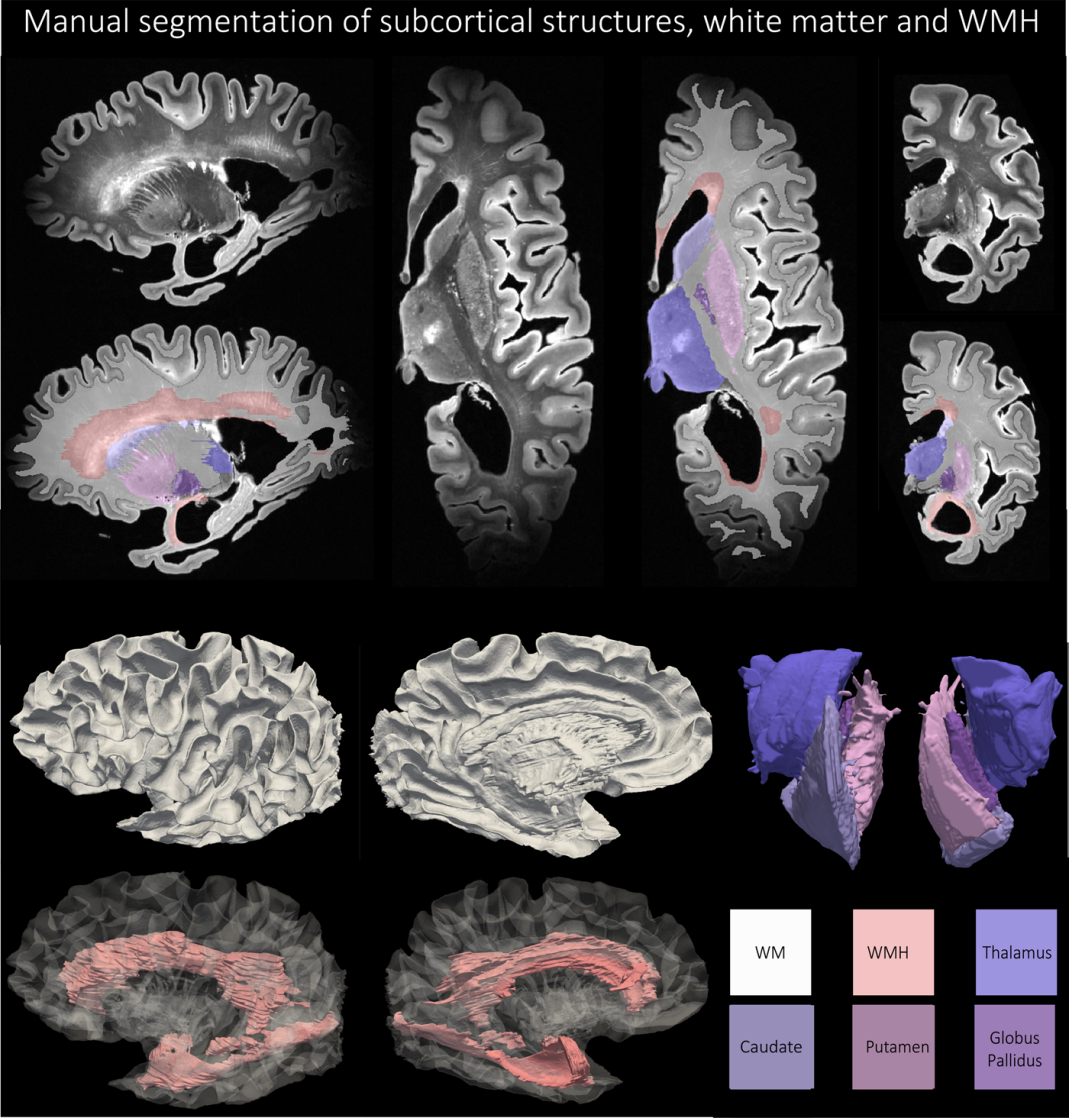
An example of manual segmentation of the thalamus, putamen, caudate,globus pallidus, and white matter hyperintensities as per theprotocol described inSections3.1.2and3.1.3fora subject with Alzheimer’s Disease and Lewy body disease (87years old).

Four subcortical gray matter structures (caudate, putamen, globus pallidus,thalamus) were manually segmented on seven specimens, selected to spanvarying pathological diagnoses (including Alzheimer’s spectrum,p-tauopathies, TDP-43 encephalopathies, LBD, cerebrovascular disease andmixed disease), the range of postmortem age and levels of cerebral atrophy,tissue quality and tearing, mineral deposition and calcification, bloodvessel size, and vascular pathology on imaging and autopsy.Figure 6shows an example manualsegmentation of the four subcortical structures. First, structures weresegmented across the axial plane based on several anatomical considerations.(1) The borders of the caudate (head, body, and tail) were defined by thefrontal horns of the lateral ventricles (cerebrospinal fluid, anteriorborder) and the internal capsule (WM, boundaries of the body).Periventricular WM disease adjacent to the head of the caudate was excludedfrom the caudate segmentation. (2) The borders of the putamen weredetermined by the globus pallidus (medial) and the claustrum (lateral). (3)The globus pallidus was annotated to include the pallidum interna andexterna, bounded by WM surrounding the thalamus and subthalamic nucleus.Mineral deposition (such as calcium) was noted by areas of heterogeneous T2hypointensities and predominantly localized to the lentiform nucleus; whereneeded, the contours of some segmentations were adjusted to include theseregions of heterogeneous signal within the lentiform structures. (4) Thethalamus was segmented as the medial gray matter bounded by the subthalamicnucleus, midbrain, mamillary bodies, corpus callosum, lateral ventricles,and caudate. After segmentation in the axial plane, volumes were edited inthe coronal and sagittal planes to ensure smoothness of the segmentationacross all three planes. Boundaries between structures (such as striationsbetween caudate and putamen) were agreed upon among authors. Manualsegmentation of the four subcortical structures was performed by author,E.C. and supervised by and edited by M.T.D. Subcortical segmentations werediscussed in consensus meetings with P.K., P.A.Y., S.R.D., and D.A.W.

#### 3.1.3 White matter hyperintensities

Nine specimens were chosen to segment WMHs across a gamut of vascularpathology with differing levels of WMH appearance, including focalsmall-vessel ischemia to intermediate periventricular and juxtacorticalpatterns to large, diffuse cerebrovascular disease. General principles forsegmentation were applied as follows. (1) segmented lesions should begenerally larger than 1 cm^3^, (2) segmentations should appear forat least 4-6 slices to be a 1 cm^3^region, and (3) segmented WMHshould include both periventricular (anterior frontal and posteriortemporal/occipital horns of the lateral ventricles) and juxtacorticallesions. This distinguished WMH from insular cortex, claustrum, basalganglia, and other gray matter structures embedded in WM. (4) WMHsegmentations generally exhibited signal intensity above a threshold of≥450-550intensity (but this was influenced by field inhomogeneity artifacts, eitherbetween images or within the same image, often at the anterior and posteriorcortical poles), given the image intensity range was normalized between0-1000. (5) WMH segmentations included T2 hyperintense perivascular spacesand cortical venules but must also include surrounding T2 hyperintense whitematter regions that occupy a region larger than 1 cm^3^. (6) WMHwas segmented primarily in axial plane and then assessed for contiguity andsmoothness in sagittal and axial planes as well as 3D renderings. Manualsegmentation was performed by the author A.E.D. and supervised and edited byM.T.D. WMH segmentations were discussed in consensus meetings with P.K.,P.A.Y, S.R.D, and D.A.W.Figure 6showsan example manual segmentation of the WMH.

#### 3.1.4 White matter

Two specimens with manually labeled subcortical structures and WMH, butnnU-Net-based automated segmentations for cortical mantle were thresholdedto obtain the WM label, which was then manually corrected by the authorA.E.D., supervised by P.K., to remove incorrectly labeled spurious voxels tofill-in holes and thereby define a clear GM/WM boundary.

### 3.2 Automated deep learning-based segmentation for cortical gray matter

We first developed an approach for labeling cortical gray matter in postmortem 7T MRI scans using the 30 patch-level cortical gray matter segmentations from sixsubjects (described inSection 3.1.1) fortraining and cross-validation. Given the exceptional performance ofconvolutional neural network (CNN) models for antemortem medical imagesegmentation (Schiffer, Spitzer, et al.,2021), our effort focused on benchmarking leading existing CNNmodels, rather than developing another model for the postmortem corticalsegmentation task. We benchmarked the following variants of popular biomedicalimage segmentation deep learning models: (1) nnU-Net (Isensee et al., 2021); four variants of AnatomyNet(Zhu et al., 2019) based onsqueeze-and-excitation blocks (Roy et al.,2018): (2) Channel Squeeze and Spatial Excitation AnatomyNet (SE);(3) Spatial Squeeze and Channel Excitation AnatomyNet (CE); (4) AnatomyNet(Vanilla); (5) Spatial and Channel Squeeze and Excitation AnatomyNet (SE+ CE); (6) 3D Unet-like network (Khandelwal & Yushkevich, 2020); (7) VoxResNet (Chen et al., 2018); (8) VNet (Milletari et al., 2016); and (9) AttentionUnet (Oktay et al., 2018).Supplementary Materialprovides architectural details of the nine deep neural networks. We use PyTorch1.5.1 and a single Nvidia Quadro RTX 5000 GPU to train the models usinguser-annotated images described inSections 3.1.1-3.1.3. To train the deep neural networks, the inputimages were standardized, and then normalized between 0 and 1.

To ensure a fair and systematic evaluation of the nine networks, we trained andevaluated the nine network architectures within the nnU-Net framework undermatched conditions (i.e., same split of data into training/validation/testingsubsets; same data augmentation strategy, same hyper-parameter tuning strategy).We first evaluated the accuracy of cortical segmentation by six-foldcross-validation on the patch-level manual segmentations. We report average DSCand average Hausdorff Distance 95th percentile (HD95) across the 30 segmentedpatches in the six-fold cross-validation experiments.

To choose the best performing model for subsequent analyses, the nine trainedmodels were then used to segment the whole hemisphere cortical mantle across asubset of the cohort (N = 36 for which the user-supervised semi-automatedsegmentations are available). We used these segmentations to compute thethickness around the 16 landmarks in these 36 subjects at the corticallandmarks. We then compared the thickness measurements to corresponding measuresobtained via user-supervised semi-automated segmentations (reference standard)using our protocol (Section 2.4). InSupplementary Figure 4,for each of the 16 landmarks, we report Spearman’s correlationcoefficient between the automated and the reference manual segmentation-basedcortical thickness measurements, and the average fixed raters intra-classcorrelation coefficient (ICC) (Shrout &Fleiss, 1979). Based on the combination of cross-validation DSCaccuracy, agreement between the automatic and manual segmentation-based corticalthickness measures, and overall visual inspection of the segmentations, a singlemodel was selected for subsequent experiments. As reported inSection 4.1, this best-performing method wasthe**nnU-Net**model.

### 3.3 Automated deep learning-based segmentation for other structures

We trained another vanilla nnU-Net model to obtain the automated segmentation ofsubcortical structures, WMH, and normal-appearing WM. In particular, for thepurpose of five-fold cross-validation studies to report DSC and HD95 scores, wetrained an nnU-Net model based on the manually labeled training data for thesegmentation of subcortical structures in seven subjects and WMH in ninesubjects; and the manually labeled WM and the automated cortical mantlesegmentations obtained from the nnU-Net model trained in the previousSection 3.2as additional labels.Supplementary Table 1lists the subjects used for training and evaluation of all the seven labels.

### 3.4 Post-hoc automated topology correction of segmentations

As shown inFigure 2, the image acquisitionsuffers from geometric distortions due to the non-linearity of the magneticgradient field and might also suffer from partial volume averaging whichgenerally introduces holes and handles in the segmentation and makes the WMsurface to lack a spherical topology. Furthermore, the opposing banks of sulcusoften appear to be fused, which makes it harder for the segmentation algorithmto correctly segment the cortex and thus might produce erroneous corticalthickness values. Hence, it is important to correct such mis-segmentations byenforcing a topology correction method. Therefore, we employ the methodsdeveloped in CRUISE: Cortical reconstruction using implicit surface evolution(Han et al., 2004) made available aspart of the “*nighres*” package, with defaultsettings (Huntenburg et al., 2018) forpost-hoc topology correction of WM surface and constraining the GMsegmentations. In particular, the method uses a fast marching-based method(Bazin & Pham, 2007), agraph-based topology correction algorithm (GTCA) (Han et al., 2002), and a topology preservinggeometric deformable model (TGDM) (Han et al.,2003) to rectify holes and handles, thus producing a WM surface witha spherical topology. Separately, the Anatomically Consistent Enhancement (ACE)method is used to provide a GM representation that has evidence of sulci whereit might not otherwise exist due to the partial volume effect. Thus, we obtaingeometrically accurate and topologically correct segmentations for the corticalGM and WM. In the current study, we apply this topology correction step on theautomated GM/WM segmentation labels obtained from the nnU-Net model trained inthe previousSection 3.3. We called thepost-hoc topology corrected model as “nnU-Net-CRUISE.”

### 3.5 Linking neuropathology ratings with morphometry

We computed Spearman’s correlation between thickness measurements obtainedfrom automated segmentations at the 16 anatomical landmarks described above withsemi-quantitative pathology ratings from approximately corresponding anatomicallocations in the contralateral hemisphere (regional p-tau score, regionalneuronal loss) and global pathology ratings (CERAD stage, Braak stage,β-amyloid).We repeat this analysis with thickness measurements obtained from user-annotatedmanual reference segmentations, and thereby test the hypothesis that similartrends would be seen between pathology correlations with automated and manualthickness measures, which, in turn, would imply that automated segmentations areviable for morphometry-based studies (Section4.4). In particular, we follow the experimental design from ourrecent work (Sadaghiani et al., 2022) fora subset of the cohort within the AD spectrum having AD as their primarydiagnosis and also diagnosis of either: LBD, PART, LATE, CBD (N = 82) outof 135. The criteria for AD continuum are based on excluding cases withdiagnoses of FLTD or non-AD tauopathy (whether primary or secondary). Finally,we normalized the WMH volumes by the corresponding WM volumes, and then computedone-sided Spearman correlation between the normalized WMH volume with regionalcortical thickness and subcortical volumes for the subjects within the ADspectrum (N = 82). We include nuisance covariates of age, sex, andpostmortem interval (PMI) in all of our analyses.

## 4 Results

### 4.1 Cortical gray matter segmentation

#### 4.1.1 Dice coefficient volume overlap and qualitative analysis

**Fig. 7.**
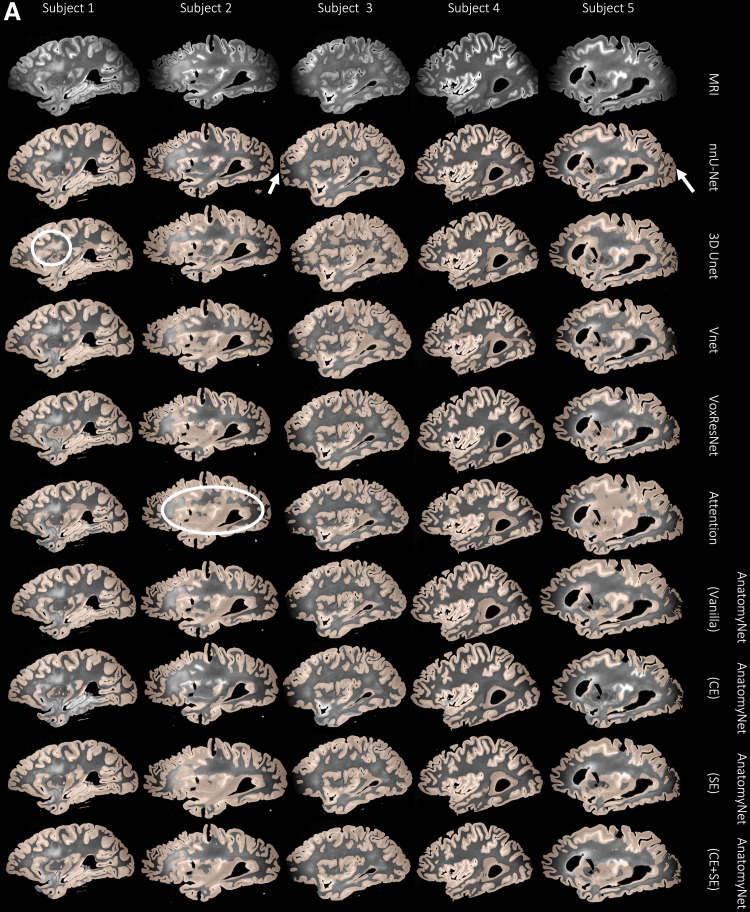

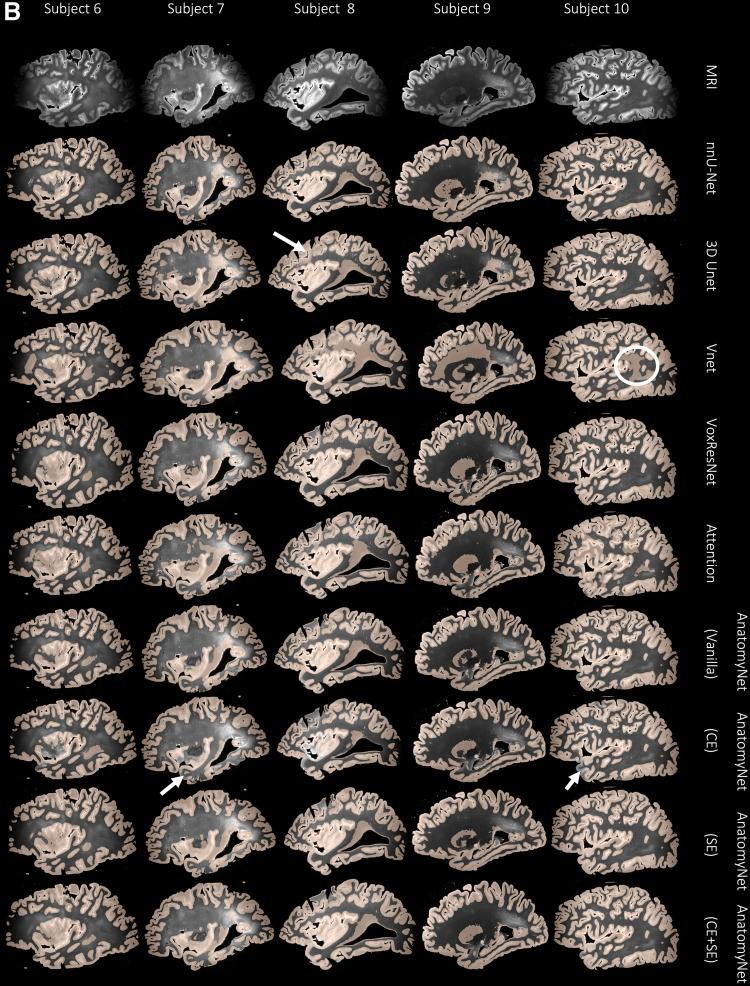
(A) For a subset of 10 subjects, each column shows an examplesagittal view of the automated cortical mantle segmentationpredicted across the whole-brain hemisphere using the differentneural network architectures. We notice that all architecturesexcept nnU-Net show either under- or over-segmentation of thecortical gray matter, which, together with the results reported inTable 3(ICC results),prompted us to select nnU-Net as the preferred model for corticalgray matter segmentation. For example, notice how networks such as3D Unet and Attention Unet incorrectly segment large chunks of WM ascortical GM (white circles). Whereas nnU-Net performs the best indifficult to segment areas such as the anterior and posteriorregions of the brain with poor MRI signal. (B) Again, notice thatAnatomyNet (CE) is not able to segment certain areas in the corticalmantle (white arrows). The 3D Unet and Vnet networks oversegment thecortical mantle into the WM. However, nnU-Net overcomes all of theselimitations.

Table 2tabulates the*patch*-level cortical gray matter segmentationperformance of the nine different networks across six-fold cross-validation.AnatomyNet and its variants attain the highest patch-level DSC, closelyfollowed by VoxResNet. The nnU-Net model has slightly lower DSC than thebest AnatomyNet model, but the difference is less that1%. However, since the patches used to train the segmentationnetworks were only sampled from select regions of the hemispheres,cross-validation accuracy on these patches is not necessarily indicative ofthe networks’ ability to generalize to other brain regions.Figure 7illustrates, that consistentlyacross our specimens, AnatomyNet and its variants are able to distinguishgray matter from white matter in high-contrast regions, but fail to segmentthe anterior and posterior regions where contrast is lower due tolimitations of the MRI coil. There is also some systematicunder-segmentation (see white arrows) of the cortex even in higher-contrastregions (see the white circled regions inFig.7). By contrast, nnU-Net clearly demarcates GM/WM boundary evenin low-contrast regions, which is remarkable considering that these regionswere not captured by the training patches.Supplementary Figure3shows the Dice scores and the HD95 scores as box plots withpairwise paired t-tests with Bonferroni correction for all the nine networkarchitectures in 2. Mainly the comparisons between VNet, the lowestperforming network, and the rest are significant. This supports our choiceof nnU-Net given that it is not significantly worse performing than any ofthe other architectures.

**Table 2.** Six-fold cross-validation dice coefficient (DSC) and Hausdorffdistance 95th percentile (HD95) scores between reference standardand automated patch-level cortical segmentations.

Deep learning method	DSC (%)	HD95 (mm)
nnU-Net	93.98 ± 5.25	0.49 ± 0.45
AnatomyNet (SE)	94.84 ± 3.84	0.45 ± 0.42
AnatomyNet (CE)	94.91 ± 3.27	0.45 ± 0.42
AnatomyNet (Vanilla)	94.86 ± 3.83	0.46 ± 0.44
AnatomyNet (CE + SE)	94.66 ± 3.79	0.47 ± 0.44
3D Unet	93.57 ± 5.22	0.58 ± 0.51
VoxResNet	94.84 ± 4.00	0.45 ± 0.42
VNet	90.84 ± 5.93	0.99 ± 0.56
Attention Unet	93.65 ± 4.91	0.62 ± 0.66

#### 4.1.2 Intra-class correlation coefficient

We compare cortical thickness (mm) at 16 cortical landmarks between automatedgray matter segmentations obtained by the nine networks and theuser-supervised semi-automated segmentation method, which serves as thereference standard.

Table 3tabulates the Average fixedraters ICC scores for all the nine networks trained as described in 3.2. Weobserve that nnU-Net (mean ICC = 0.72) is clearly the best among thenine patch-based models. The final nnU-Net-CRUISE model which has beentopologically corrected as described inSections 3.3and3.4slightly exceeds the patch-based vanilla nnU-Net (mean ICC of 0.73) onaverage. The variants of AnatomyNet have mean ICC values of, Vanilla: 0.40,CE: 0.47, CE+SE: 0.40, and SE: 0.34. We observe that the variants ofAnatomyNet were the top performing models when evaluated using DSC scores atpatch level, but did not generalize to robustly segment the entire corticalmantle; and thereby failed to show good correlation of regional corticalthickness when compared with the reference standard reference corticalthickness. The other four models also had very low ICC values: VoxResNet:0.45, VNet: 0.28, 3D Unet: 0.47, and Attention Unet: 0.35.

**Table 3.**
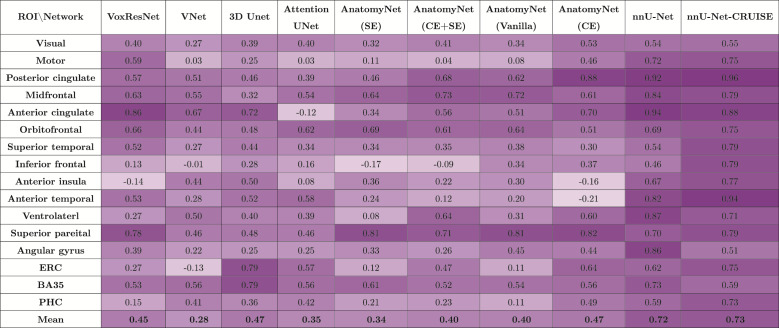
Average fixed raters intra-class correlation coefficient (ICC) scoresfor the regional cortical thickness measurements between automatednnU-Net and reference standard manual segmentations for all the nineneural network architectures, and the topologically correctednnU-Net-CRUISE segmentations.

Across each row, each cell is color coded, with darker shadesindicating higher ICC value.

The Bland-Altman plots inFigure 8showstrong agreement between reference standard and post-hoc topologicallycorrected automated nnU-Net segmentation-based thickness measurements forthe 16 cortical landmarks. Furthermore,Supplementary Figure4shows that 13 out of the 16 regions have correlationcoefficient (r) greater than 0.6. We also observe high ICC scores, with 12regions having ICC greater than 0.7. These results confirm that automatedsegmentations are accurate to give desirable cortical thicknessmeasurements.

**Fig. 8.**
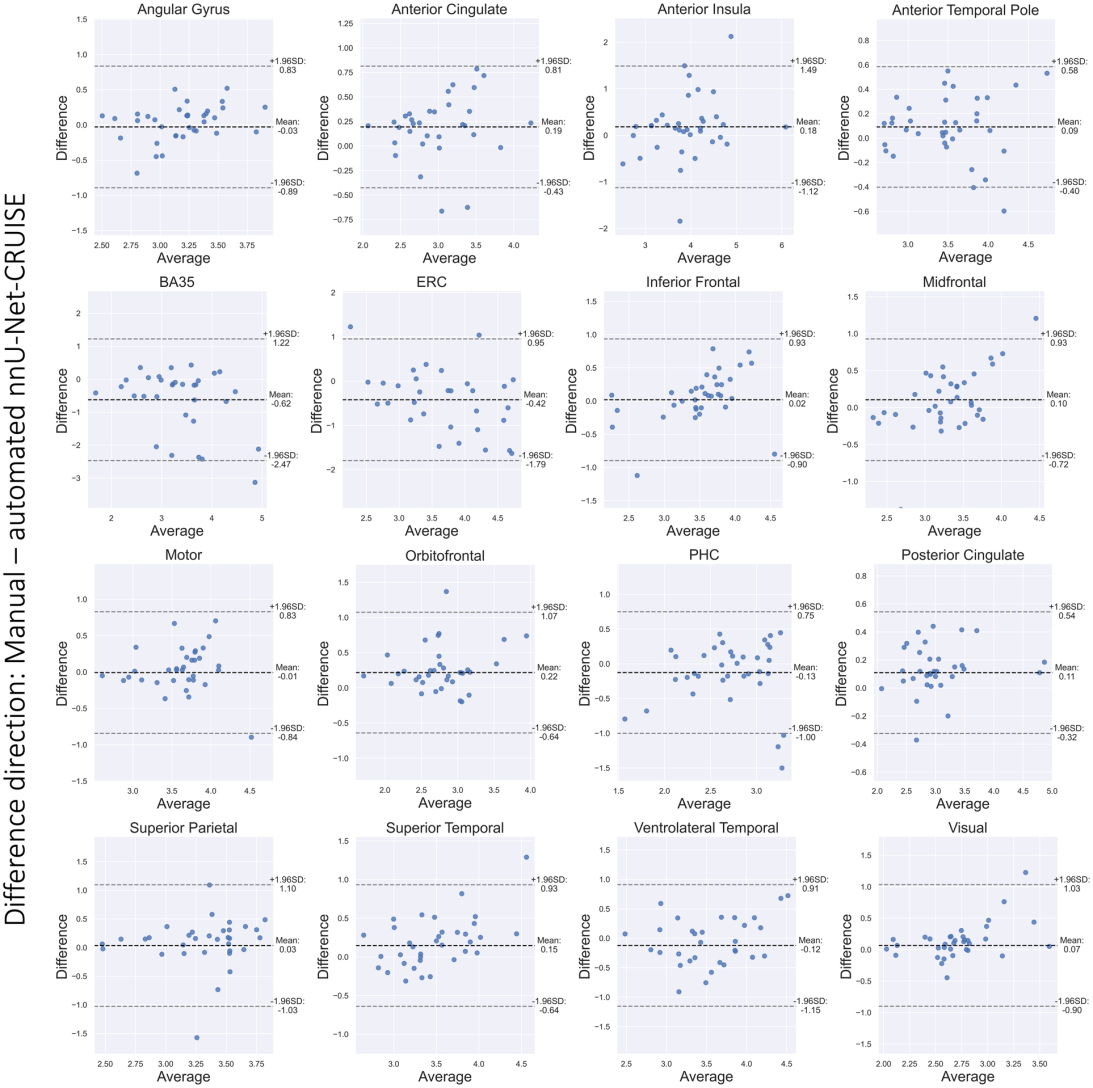
Bland-Altman plots for the cortical regions. We observe goodagreement between the manual and nnU-Net-CRUISE automatedsegmentations-based thickness (mm), which supports the hypothesisthat topologically corrected deep learning-based automatedsegmentations are reliable for morphometric measurements. Note thatthe highlighted differences are in the direction: manual-automatedsegmentations.Supplementary Figure 3shows the correspondingcorrelation plots.

**Fig. 9.**
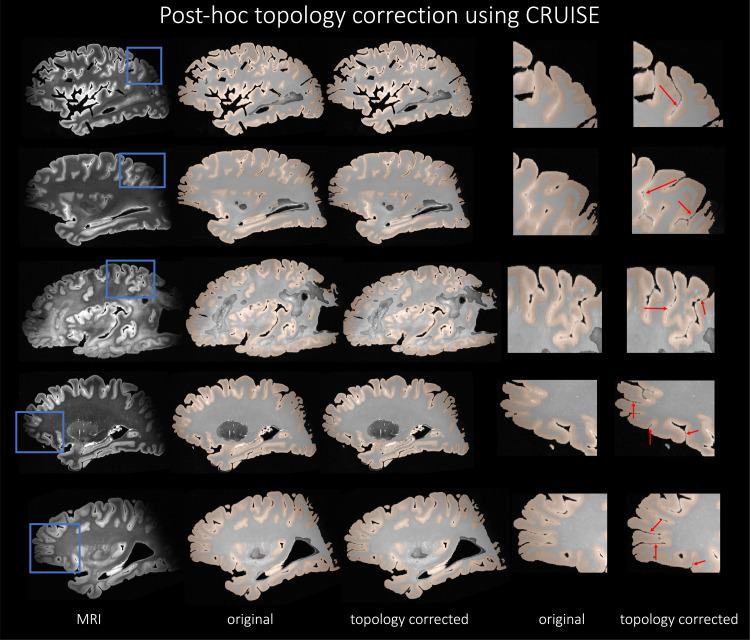
nnU-Net-CRUISE. Topologically corrected automated nnU-Netsegmentations of cortical gray matter and white matter shown insagittal view for five subjects. The first columns shows the MRIslice, the automated nnU-Net segmentation before and after topologycorrection step (nnU-Net-CRUISE) are shown in columns 2 and 3,respectively. Columns 4 and 5 show the zoomed-in area, with the redarrows indicating the regions where topology correction improves thesegmentation.

Therefore, based on quantitative evaluation in terms of DSC and HD95 scores,ICC values, and the qualitative visual inspection of the segmentations forthe different neural network architectures, we conclude that nnU-Net-CRUISEis the best performing model.Figure 9shows the post-hoc topology corrected automated nnU-Net segmentations ofcortical gray matter and white matter shown in sagittal view for fiverandomly chosen subjects. The first columns shows the MRI slice, theautomated nnU-Net segmentation before and after topology correction step areshown in columns 2 and 3. Columns 4 and 5 show the zoomed-in area, with thered arrows indicating the regions where topology correction improves thesegmentation. We notice that the opposite banks of sulci are no longer fusedand in fact well demarcated after correcting for topology. Thesesegmentations will therefore provide more reliable and accurate estimates ofcortical thickness.

### 4.2 Other structures: subcortical, WMH, and WM segmentation

**Fig. 10.**
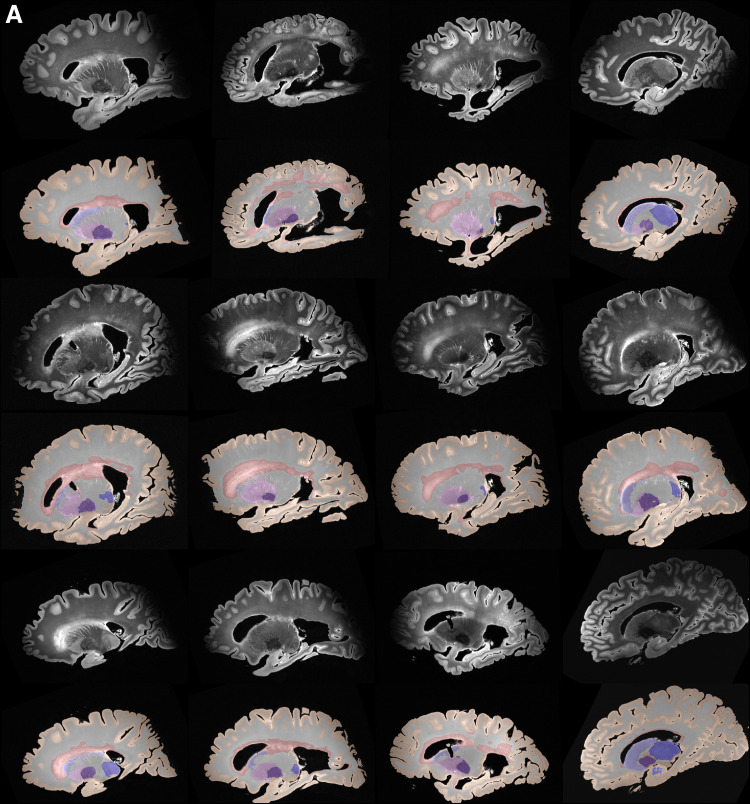

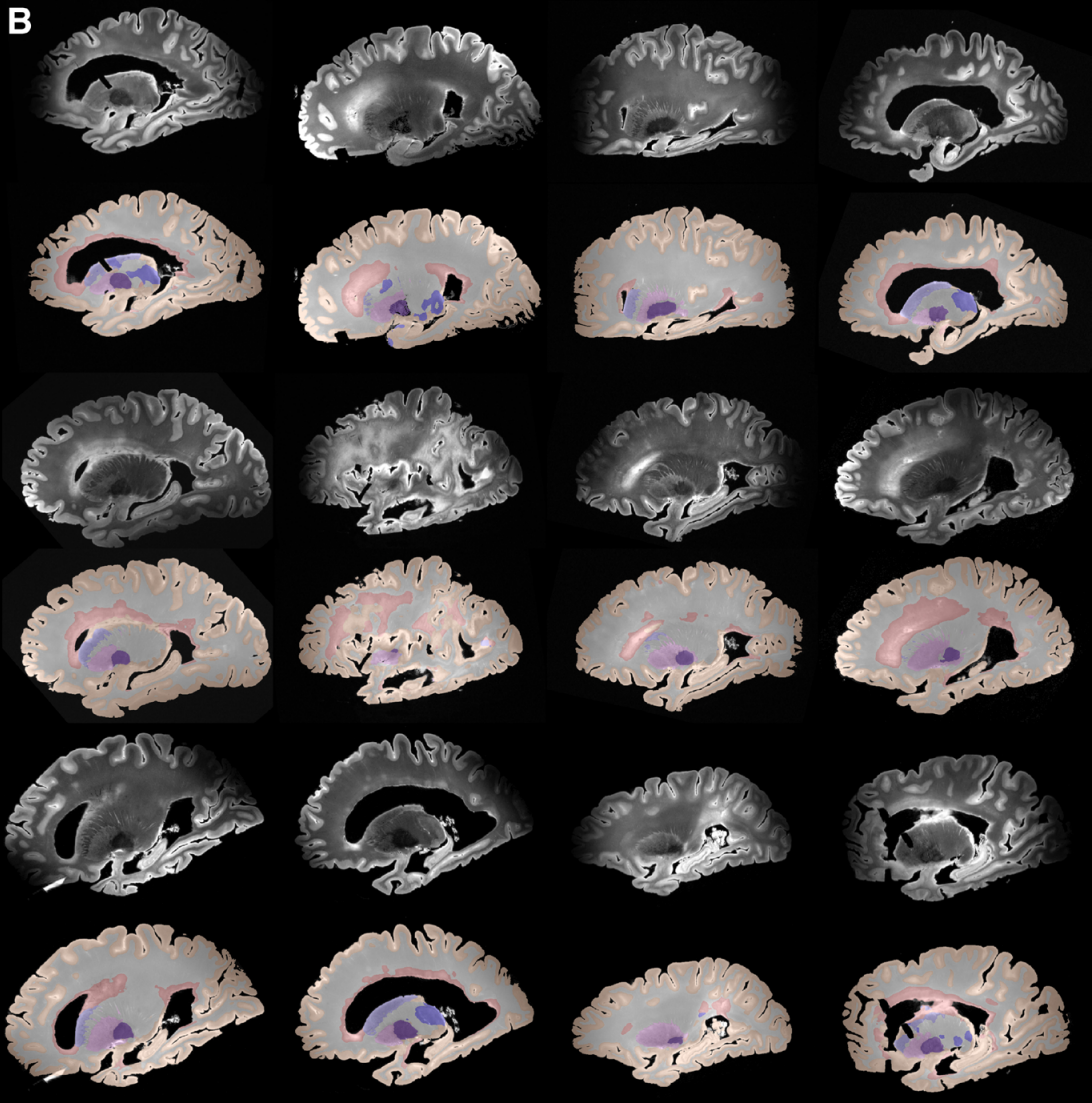

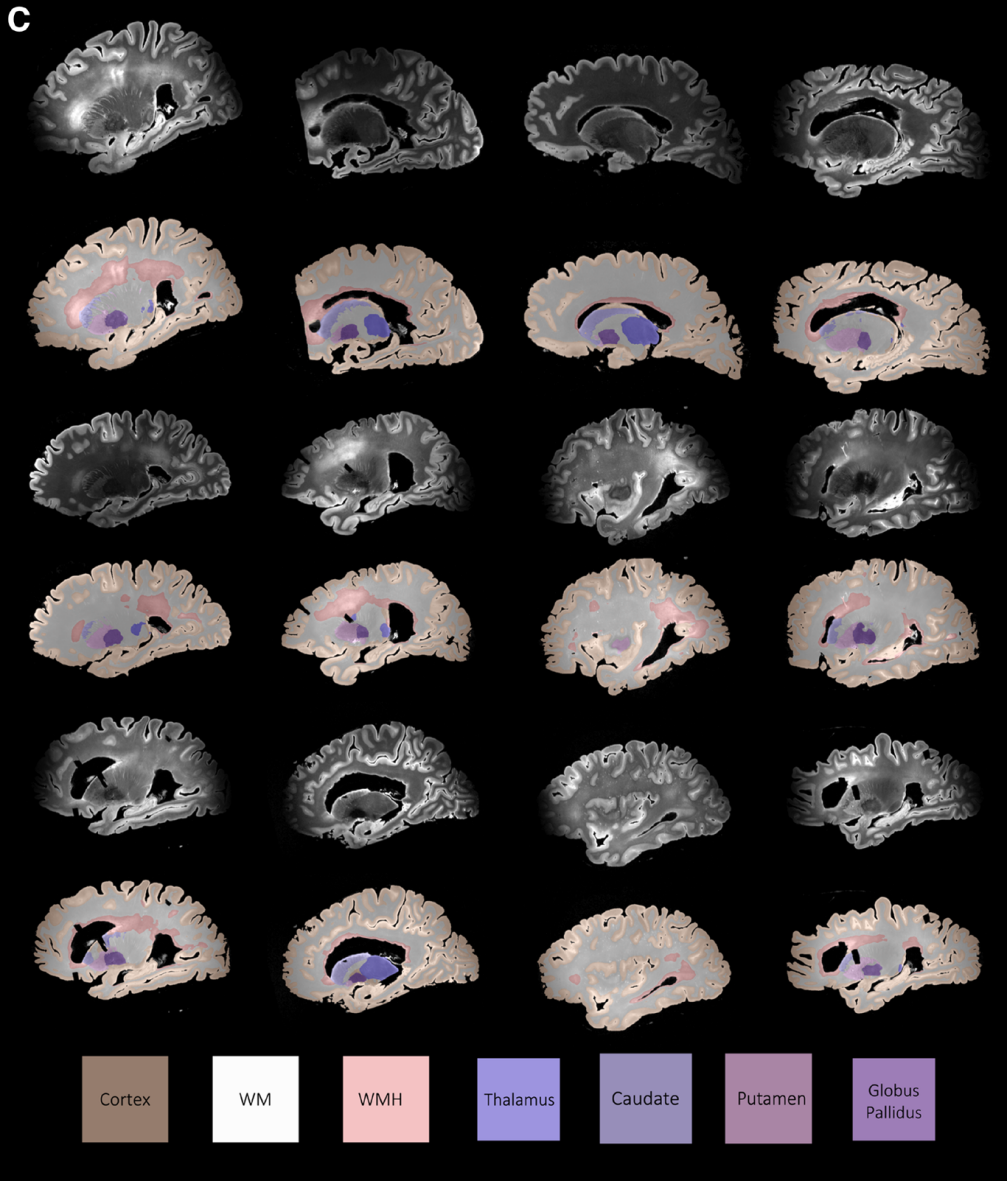
(A, B, C) Automated segmentations by nnU-Net of cortical gray matter,WMH, WM and the four subcortical structures for a sample of 36 subjectsin the cohort.

Based on the superior performance of nnU-Net, as explained in the previoussection, we employ nnU-Net to perform the multi-label segmentation for WMH,caudate, putamen, globus pallidus, thalamus, and WM. The mean DSC scores for thefour subcortical structures and WMH across all leave-one-out cross-validationexperiments were: WMH: 79.70 %, caudate: 88.18 %, putamen: 85.20 %, globuspallidus: 80.12 %, and the thalamus: 87.29 %. Note that currently, we do nothave a large sample size to perform cross-validation evaluation on the WM, dueto the time-consuming process of manually correcting the segmentations.Obtaining manual WM label is beyond the scope of the current study.Figures 10and11show qualitative results for all the segmentedstructures in 2D and 3D respectively for the 36 subjects used for quantitativeevaluation in the previous section, in sagittal view.

**Fig. 11.**
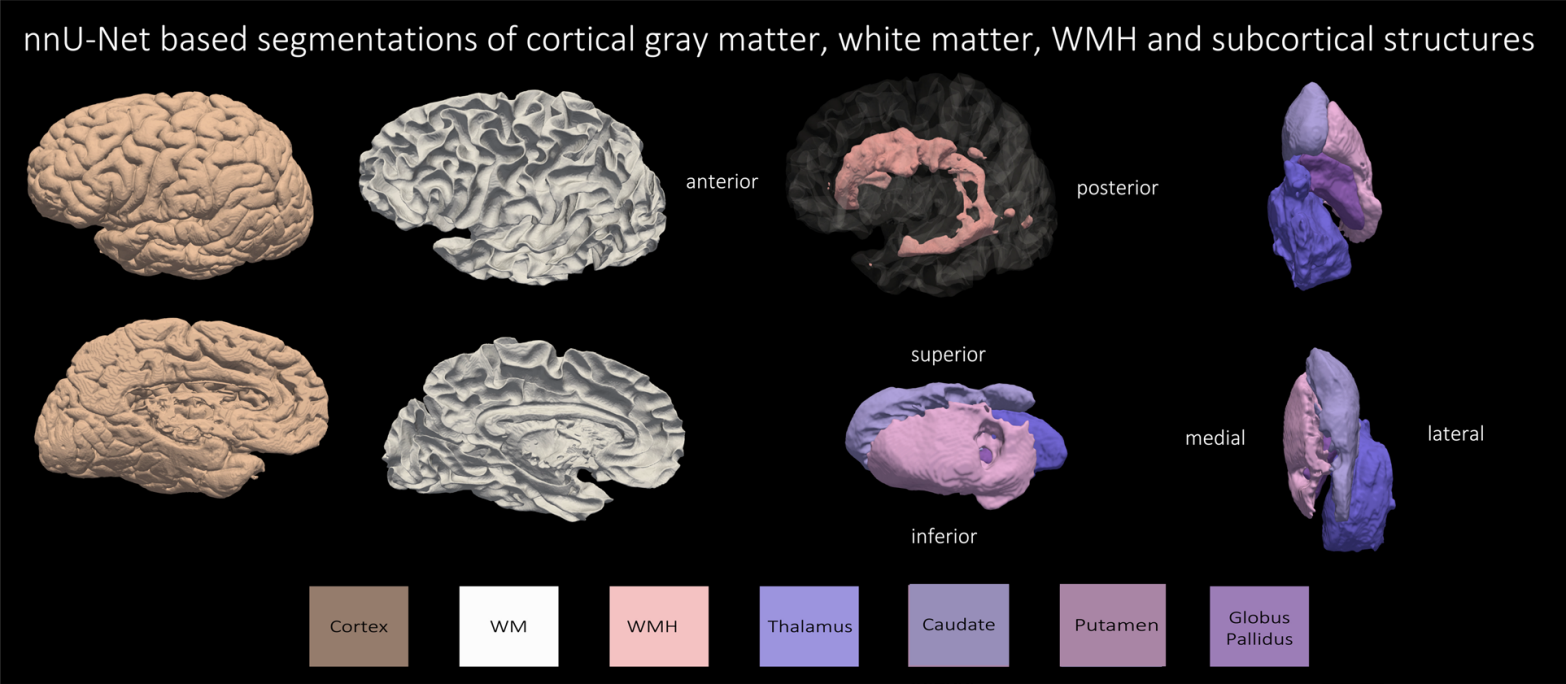
Three-dimensional renderings of automated segmentations by nnU-Net ofcortical gray matter, WM, WMH and the four subcortical structures forthe subject with primary age-related tauopathy and cerebrovasculardisease.

### 4.3 Generalization to other imaging sequences and protocols

**Fig. 12.**
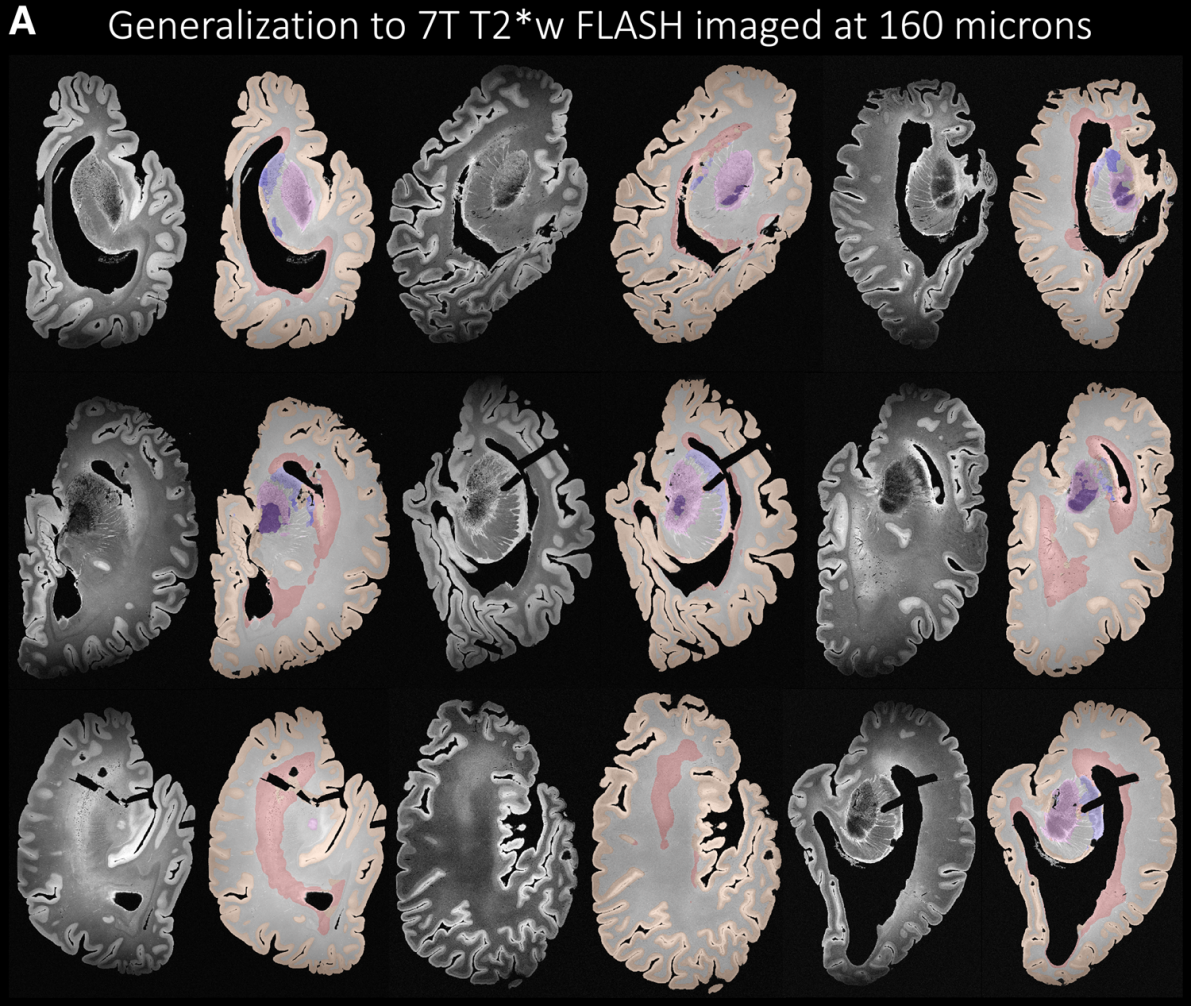

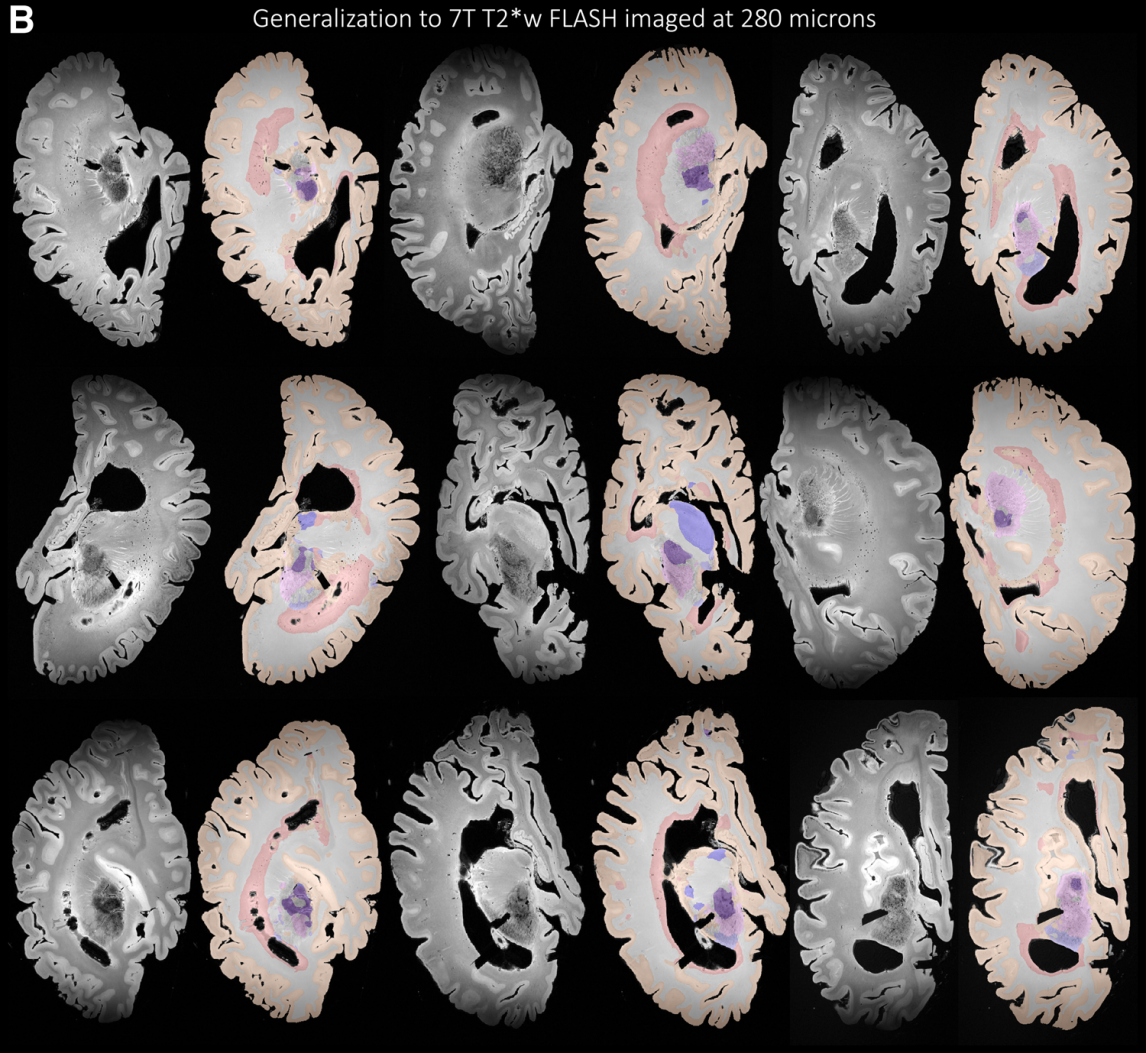
Generalization to other unseen imaging sequences. (A) The nnU-Netarchitecture trained on 7 T T2w images at 0.3 x 0.3 x 0.3 mm^3^generalized well to images acquired at 7 T T2*w at a highresolution of 0.16 x 0.16 x 0.16 mm^3^. (B) The nnU-Netarchitecture trained on 7 T T2w images at 0.3 x 0.3 x 0.3 mm^3^generalized well to images acquired at 7 T T2*w at a highresolution of 0.28 x 0.28 x 0.28 mm^3^.

Further highlighting the strong generalization properties of nnU-Net,Figure 12illustrates that the nnU-Net modeltrained on 7 T 0.3 x 0.3 x 0.3 mm^3^T2w images is able to generalizewell to MRI sequences and resolutions unseen during training. In particular, weevaluate the trained model on 7 T T2*w GRE FLASH sequence postmortemimages acquired (Tisdall et al., 2021) at0.28 x 0.28 x 0.28 mm^3^(N = 13) and 0.16 x 0.16 x 0.16mm^3^(N = 73) resolution as shown qualitatively inFigure 12. We observe good generalizationperformance for GM, WM and WMH but observe some under-segmentations in thesubcortical structures for both the 160 and 280 micron sequences. Currently, weonly provide qualitative assessment on a representative sample of the FLASHimages. The limitation lies in quantitative assessment (beyond the scope of thecurrent study) in terms of Dice score and ICV which is currently not possibledue to lack of reference manual segmentation.

### 4.4 Morphometry associations with underlying neuropathology

#### 4.4.1 Regional cortical atrophy correlation patterns with underlying ratings

We correlate regional cortical thickness measures derived from nnU-Net-CRUISE gray matter segmentation with corresponding regional ratings of p-tau pathology and neuronal loss density, amyloid- β ratings, CERAD, and Braak staging as explained in Section 3.5 . Table 4 tabulates the correlation between the regional cortical thickness and the pathology ratings for measurements based on automated nnU-Net-CRUISE segmentations for the subjects within the AD continuum (N = 82). For the automated nnU-Net segmentation-based regional cortical thickness measurements, we observe significant negative one-sided Spearman’s correlation which survived Bonferroni multiple corrections (corrected *p* -value <0.05), controlling for age, sex, and PMI:

- in posterior cingulate (*r*= -0.46,*p*= 0.0001), mid-frontal(*r*= -0.441,*p*= 10^−5^), superior temporal(*r*= -0.325,*p*= 0.002), and entorhinal cortex (*r*= -0.429,*p*= 0.0001) withamyloid-β;
- in posterior cingulate (*r*=-0.445,*p*= 0.0002), midfrontal(*r*= -0.327,*p*= 0.0018) and entorhinal cortex (*r*= -0.522,*p*=10^−5^), and parahippocampal cortex(*r*= -0.328,*p*= 0.0018) with Braak staging;
- in posterior cingulate (*r*=-0.419,*p*= 0.0004) and entorhinalcortex (*r*= -0.335,*p*= 0.0016) with CERAD rating;
- in posterior cingulate (*r*=-0.357,*p*= 0.0024), midfrontal(*r*= -0.383,*p*= 0.0003), and entorhinal cortex (*r*= -0.348,*p*= 0.0011) withregional p-tau rating;
- in entorhinal cortex (*r*= -0.414,*p*= 0.0001) with regional neuronalloss rating.

**Table 4.**
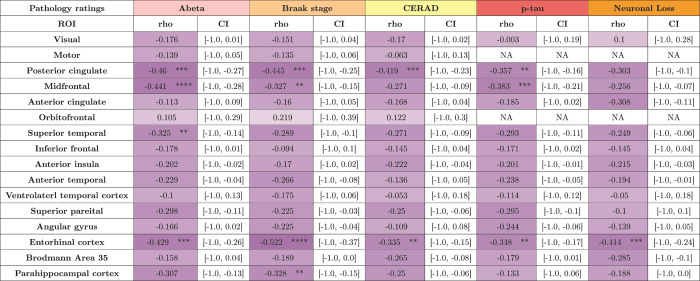
Morphometry associations with underlying neuropathologymeasurements.

Shown is the one-sided Spearman’s correlation, controllingfor age, sex, and PMI, between regional cortical thicknessmeasures derived from topologically corrected nnUNet-CRUISE graymatter segmentation with corresponding regional ratings of p-taupathology, neuronal loss density, and global ratings ofamyloid-β, CERAD, and Braak staging. Each cell is colorcoded, with darker shades indicating more negative correlations.The asterisk indicates that the test survived Bonferronimultiple testing correction (Bonferroni, 1935). CI indicates 95% confidenceinterval. *:0.01 <*p *≤0.05; **:0.001 <*p*≤ 0.01; ***:0.0001 <*p *≤ 0.001;****:0.00001 <*p*≤ 0.0001.

Supplementary Table3shows the one-sided Spearman’s correlation (controllingfor age, sex and PMI) between the cortical thickness measures derived fromthe topologically corrected nnU-Net-CRUISE gray matter segmentation with thecorresponding medial temporal lobe (MTL) ratings of p-tau pathology andneuronal loss density as the MTL is a region linked to earlyneurodegeneration in Alzheimer’s disease. The MTL pathology ratingsare computed by taking the average pathology of the entorhinal cortex, CA1and subiculum, and the dentate gyrus. We notice significant results in theentorhinal cortex (*r*= -0.53,*p*= 10-5) with the MTL p-tau rating; and in posterior cingulate(*r*= -0.396,*p*=0.0008), entorhinal cortex (*r*= -0.47,*p*= 10-5), and BA35 (*r*=-0.321,*p*= 0.0026) with the MTL neuronal lossdensity.

Separately,SupplementaryTable 4tabulates the morphometry associations with theunderlying neuropathology for the AD continuum (N = 21) subjects fromthe subset of the cohort which have the manual segmentations (N = 36)as described inSection 3.2. Weobserve that the analysis based on manual segmentations shows a similartrend as the nnU-Net-CRUISE automated segmentations, suggesting thatautomated segmentations obtained from the developed pipeline providemeaningful associations with the underlying neuropathological measurements,are thus reliable for validation of clinical ratings, and can act assurrogates for the time-consuming user-supervised segmentations. Finally,SupplementaryFigures 7-13depict all the correlations as a plot along with thep-values to appreciate the data distribution.

#### 4.4.2 Normalized WMH volume correlation patterns with regional cortical thickness and subcortical volumes

Table 5compares the correlationbetween the regional cortical thickness and subcortical volumes with thenormalized WMH (WMH volume divided by the total WM volume) volume formeasurements based on automated nnU-Net-CRUISE for the subjects in the ADcontinuum (N = 82). We observed significant negative one-sidedSpearman’s correlation which survived Bonferroni multiple correctionsin posterior cingulate (*r*= -0.448,*p*= 0.0001) and midfrontal cortex(*r*= -0.325,*p*= 0.0019)with normalized WMH volume for the automated nnU-Net segmentation-basedregional thickness measurements; and in caudate (*r*=-0.399,*p*= 0.0001) and thalamus (*r*= -0.352,*p*= 0.0007) for the subcorticalstructures when correlated with the normalized WMH volume. All the testswere controlled for age, sex, and PMI.Supplementary Figures14–16depict these correlations as a plot along with thep-values to observe the data distribution.

**Table 5.**
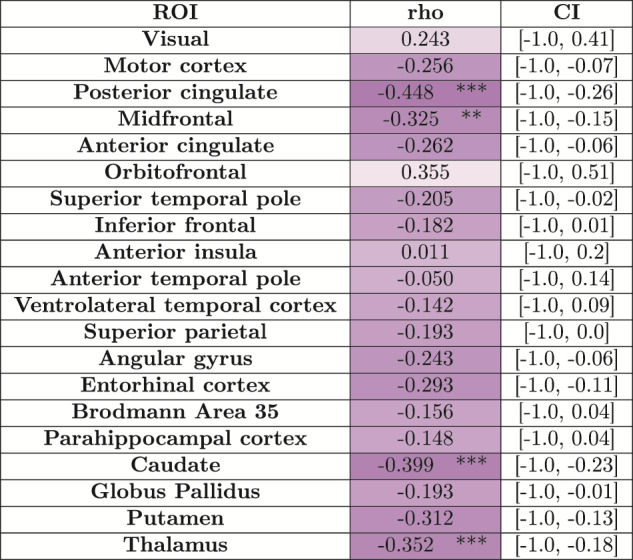
White matter hyperintensities volume correlations.

Shown is the one-sided Spearman’s correlation betweennormalized WMH volume (obtained by dividing the WMH volume withthe corresponding WM) with regional cortical thickness based onnnU-Net-CRUISE. For the four subcortical structures, partialone-sided Spearman’s correlation, with uncorrectedp-value in brackets, is shown. All the tests were controlled forage, sex, and PMI. The asterisk indicates that the test survivedBonferroni multiple tests correction. Each cell is color coded,with darker shades indicating more negative correlations. CIindicates 95% confidence interval. *:0.01<*p≤*0.05; **:0.001<*p≤*0.01;***:0.0001<*p≤*0.001; ****:0.00001<*p≤*0.0001.

## 5 Discussion

### 5.1 Segmentation pipeline

To our knowledge, the current study is the most comprehensive assessment ofautomated segmentation of 7 T postmortem human brain MRI. Our evaluation isperformed in a large cohort of 135 brain specimens with a range ofneurodegenerative pathologies, and focuses on multiple tasks: cortical graymatter segmentation, subcortical gray matter structure segmentation, as well aswhite matter and WMH segmentation. For cortical segmentation, we evaluated ninedeep learning architectures using direct metrics of segmentation accuracy(cross-validation DSC), derived morphological metrics (ICC of regional corticalthickness with the reference standard; comparison of associations withpathology), and visual assessment.

Our paper stands apart from recent work on automated segmentation of postmortembrain MRI, which has either been performed in lower-resolution 3T MRI scans(Mancini et al., 2020), or in smallerhigh-resolution datasets (Jonkman et al.,2019).Mancini et al. (2020)used FreeSurfer (Fischl, 2012) and aBayesian modeling technique, SAMSEG (Puonti etal., 2016), to map a single postmortem specimen imaged at 3 T but didnot evaluate on higher resolution 7 T. In addition to segmenting the corticalgray matter,Mancini et al. (2020)parcellate the cortex into anatomical regions. A separate study (Kotrotsou et al., 2014) also segmented the entirehemisphere but applied to a smaller dataset of 7 subjects with a slice thicknessof 1.5 mm and imaged at 3 T. Both these methods relied on multi-atlas basedimage segmentation which is dependent on registration between high-resolutionpostmortem and low-resolution antemortem atlases. Registration betweeninter-modality postmortem and antemortem currently remains challenging,especially for higher-resolution 7T postmortem MRI (Casamitjana et al., 2021;Casamitjana et al., 2022;Daly et al., 2021). Other recent studies onpostmortem human brain morphometry (Adler et al.,2014,^2018^;Augustinack et al., 2014;DeKraker et al., 2018,^2021^;Iglesias etal., 2015;Ravikumar et al.,2021;Wisse et al., 2021;Yushkevich et al., 2021) focused onspecific areas such as the hippocampus or the MTL, and relied on manualsegmentation to guide inter-subject registration and atlas generation.

Our evaluation demonstrates that deep learning-based segmentation pipelines,particularly nnU-Net, can generate high-quality segmentations of cortical graymatter, subcortical structures, normal-appearing white matter, and white matterlesions even with very limited training data. With inference time of around 15minutes on a CPU, our pipeline represents the first step towards fast,automated, and reliable brain mapping of high-resolution postmortemwhole-hemisphere MRI. Our nnU-Net based pipeline generalized well to areas oflow contrast unseen during training, as well as to other MRI protocols, andresolutions. Furthermore, our pipeline imposed post-hoc constraints on theautomated segmentations to produce geometrically accurate and topologicallycorrect segmentations using deformable surface-based methods, which have showntremendous success in the antemortem literature. Therefore, we were able tocorrectly label the challenging boundary deep sulci and the cortical graymatter, leading to more reliable cortical thickness estimates. Moreover,thickness measures derived from the deep learning-based automated segmentationsconcur with the reference standard, and similar associations between thicknessand pathology are detected using automatically derived and reference standardthickness measurements, albeit with the latter usually having higher effectsizes. This suggests that fully automated cortical thickness analysis isfeasible for postmortem MRI. Indeed, with further improvements to accuracy(e.g., a larger training set covering more of the hemisphere), automatedpostmortem segmentation may make the labor-intensive and subjectivesemi-automated approach to cortical thickness measurement unnecessary. Thus, thestudy suggests the feasibility of a fully automated group-wise corticalthickness analysis in postmortem MRI analogous to the way FreeSurfer is used forantemortem MRI morphometry, which forms the basis of our future researchdirection.

The limitations of the current pipeline include a relatively small wholehemisphere cortical gray matter segmentation training set which limited ourability to use direct metrics such as DSC to evaluate overall segmentationaccuracy. Indeed, the method that performed best in terms of cross-validationDSC (AnatomyNet) performed worse in areas unseen during training than nnU-Net.To address this limitation, in future work we plan to train the method on manualsegmentations of whole hemispheres; however, generating such a dataset willrequire a significant additional investment in time and effort. A limitation inthe cross-validation experiment comparing the nine network architectures (Table 2) is that the 30 patches were notstratified by specimen during cross-validation, that is, patches from the samespecimen appeared in both training and test subsets, which may have led toslight overestimation of Dice coefficients inTable 2. However, the patches within each subject were verydifferent in terms of anatomical location, and crucially, the main validationexperiment in this paper (ICC between expert-guided and automatic corticalthickness measurements inTable 4) isfree of such data leakage. We still rely on manually placed landmarks to measurethickness in specific anatomical regions, and we do not show 3D maps ofthickness as is common in antemortem morphometry studies. For example, thevariability in the placement of the BA35 landmarks may explain our observationof the stronger thickness/pathology associations in the ERC than in BA35, eventhough BA35 is thought to have early tau pathology. Moreover, we noticed thatfour regions (BA35, superior parietal, angular gyrus, and the visual cortices)have the lowest ICC scores between the manual reference and automatednnU-Net-CRUISE segmentations. InSupplementary Figure 6, we show a sample segmentationwhere we observe some discrepancy between the manual reference and automatednnU-Net-CRUISE segmentations. Some of the possible reasons for thesediscrepancies are in fact: over- or under-segmentation, MRI artefacts, or, asmentioned above, the lack of precision in the placement of landmark on which thethickness computation is directly dependent.

Due to limited availability of reference standard segmentations, parcellating thebrain into subregions, as in done in most antemortem MRI, currently remains achallenge. We intend to address this limitation by semi-manually annotating thebrain into different cortical and subcortical structures, guided by anatomicalpriors derived from antemortem MRI studies. We could then develop techniques forgroupwise normalization studies by building a template for postmortem MRI.Towards this goal, we are currently developing deep learning-based methods forregistration between postmortem and antemortem MRI. Our work is limited to ADRD,but in future we plan to evaluate our methodological pipeline on a cohort ofnon-demented specimens obtained from a separate postmortem dataset (Boon et al., 2019;Frigerio et al., 2021;Jonkman et al., 2019).

Certain limitations of the postmortem imaging should be noted.Wisse et al. (2017)compared the cortical thicknessof the MTL substructures between antemortem and postmortem; and also postmortem(3T) and postmortem (9.4T) MRI. They found differences in thickness on differentMRI scans and attributed to the various factors such as: (1) difference in theactual size between the antemortem and postmortem tissue is due to an actualdifference in size as studies have suggested that tissue changes may occurduring or after death since the agonal state causes hypoxia and ischemia whichresults in brain swelling. (2) an increase in size could result from brainextraction, for example, by a relief of intracranial pressure after autopsy. (3)formalin fixation could cause the underlying differences by shrinking the brainafter several weeks.

Separately, at the moment, we do not have a way to quantitatively assess theaccuracy of the T2*w FLASH images. In follow-up work, we plan to collectreference data by manually correcting the mis-segmentations and then re-trainthe deep learning model in a few-shot learning setting to achieve a boost insegmentation performance for T2*w FLASH MRI. Then, we will quantitativelyassess the segmentation performance for the said generalization procedure usingreference user-supervised manually corrected reference segmentation.

### 5.2 Neuropathology associations

With the help of the proposed segmentation and morphometry pipeline, we were ableto conduct studies of postmortem MRI that have not been possible before: forexample, we replicated some of the findings fromSadaghiani et al. (2022), and drew associations between WMH volume,cortical thickness, and subcortical volumes. Here, we discuss some of theinteresting findings. Strong postmortem image analysis frameworks allow us tobetter understand the distinct roles and degrees by which antemortem pathologyaffects neurodegeneration. Prior work shows howamyloid-β, p-tau, TDP-43, etc. have differential influences on atrophy(Dugger & Dickson, 2017;Matej et al., 2019;Negash et al., 2011;Robinson et al., 2018), and an automated pipeline and dataset asshown here can bolster the quantitative interrogation of these open questions.It will allow future work to study links between macro structure and other localprocesses beyond pathology, including inflammatory markers, gene expression,etc. We also note that measuring WMH in postmortem imaging adds value tohistological studies, as we have less clear measures of vascular burden withtraditional autopsy, which will allow probing of WMH for better understandingtheir pathologic correlates given their non-specific nature.

The current study demonstrates the associations between regional corticalthickness measurements with the underlying semi-quantitative neuropathologicalratings for the AD cohort. Negative correlations between p-tau and corticalthickness were found to be significant in angular gyrus and midfrontal regions,which is in line with previous research in antemortem (Das et al., 2019;Harrison et al., 2021;LaPoint etal., 2017;Whitwell et al.,2018;Xia et al., 2017) andpostmortem*in situ*MRI (Frigerio et al., 2021) studies. Tau pathology is concurrent withneuronal loss in ADRD (Dawe et al., 2011;Jack Jr et al., 2018;Ohm et al., 2021) and the loss of neurons is likely akey source of cortical atrophy. We observed that cortical thickness showedsignificant negative correlation with neuronal loss in BA35 and entorhinalcortex, regions where p-tau pathology has predicted the atrophy rate (La Joie et al., 2020;LaPoint et al., 2017;Xie et al., 2018) in antemortem studies. Significant negativecorrelations were observed between Braak staging and cortical thickness inmidfrontal, ERC and BA35, regions consistent with high p-tau uptake in positronemission tomography (PET) imaging with cortical thickness on MRI. Tau pathologyin Braak regions plays an important role in cortical atrophy and cognitivedecline during the course of AD. Similar findings are reported for globalcortical thickness with*in situ*postmortem MRI inFrigerio et al. (2021). The relationshipbetweenamyloid-βand neurodegeneration is thought to be rather indirect (Gómez-Isla & Frosch, 2022;Jack Jr et al., 2018). Nevertheless, we didfind strong negative correlations between thickness andamyloid-βin midfrontal, inferior frontal, and ventrolateral temporal cortex, brainregions implicated in working memory capacity (Barbey et al., 2013;Chiou &Ralph, 2018). Our observation of a significant negative correlationof CERAD scores with cortical thickness in the superior parietal region isconsistent with previous studies relating CERAD with cortical thickness (Paajanen et al., 2013;Santos et al., 2011).

Lastly, WMH has been implicated in age-related cognitive decline and AD, which ischaracterized by atrophy in the cortical mantle and the MTL (Dadar et al., 2022;Du et al., 2005;Rizvi et al.,2018). In our study, we observed significant negative correlationsbetween normalized WMH and thickness in posterior cingulate and superiortemporal regions. Previous work (Reijmer et al.,2015) showed that the disruption of structural and functionalconnectivity has an impact on executive functioning and memory among individualswith high WMH volume. To this point, our study found that subcortical atrophywas significantly negatively correlated with WMH volume in caudate and thalamus,suggesting more global effects on brain volume.

In our dataset, among the 135 specimens, 82 had AD pathology with existence ofco-pathologies. Future studies may apply this dataset and pipeline to helpdisentangle the differential contributions of unique pathologies to individualatrophy patterns. Separately, we are aware that the pathology measures and MRIsegmentation-based measures were obtained from contralateral hemispheres, whichcould potentially weaken the observed associations. But pathology in AD isusually largely symmetrical between the hemispheres, and therefore leaves lessroom for biases in the observed correlations, as claimed in a recent study(Ravikumar et al., 2021) which showedthat correlations between MTL thickness maps and both contralateral andipsilateral semi-quantitative p-tau pathology scores did not detectsubstantially different correlation patterns. Overall, the fact that strongthickness-pathology associations were observed for many brain regions even inthis sub-optimal setting, supports our overall hypothesis that thicknessmeasures derived using the proposed automatic pipeline are suitable for brainmorphometry-pathology association studies. In future work, we plan to examineassociations between these thickness measures and ipsilateral quantitativemeasures of pathology matched via multi-modality image registration.

Another limitation is that our study relies on semi-quantitative measures ofneuropathology, which are subjective and might not reflect a linear pathologyburden. We are currently in the process of obtaining neuropathology measurementsfrom the same hemisphere histology, and developing machine learning-basedquantitative pathological ratings to further validate our work. The currentstudy provides support for future work to use larger datasets and quantitativepathology measures to describe the contribution of multiple pathologies to brainmorphology in neurodegenerative diseases. But, overall we observe a similartrend as described in our recent work (Sadaghiani et al., 2022), which looked at regional corticalthickness with p-tau burden. These limitations could be avoided by expanding ouranalysis to a larger dataset, which we are actively working towards.

Lastly, we should mention that our postmortem imaging project was launchedshortly before the COVID-19 pandemic, which greatly interfered with our abilityto maintain consistency in formalin fixation. This has been addressed in ourCenter’s more recent autopsies, where we aim for consistent 60 daysfixation. Like other limitations, we hypothesize that if formalin fixation hadbeen more consistent, that would only lead to stronger associations betweenstructure and pathology. The plot inSupplementary Figure 5shows the mismatch in thickness(in mm) between automated (nnU-Net-CRUISE) and manual reference segmentationsversus fixation time (in days). The plot does not reveal a systematicrelationship between fixation time and thickness mismatch, neither in terms ofbias nor in terms of variance. This is consistent with the literature that showsrelative plateauing of T2 values in postmortem MRI after initial 1-2 monthsfixation (Dawe et al., 2009). We concludethat the large variation in fixation times in our study did not significantlyaffect the thickness computation based on the obtained segmentations.

## 6 Conclusion

While there is increased interest in using high-resolution postmortem MRI of thehuman brain for discovering associations between brain structure and pathology,automated tools for the analysis of such complex images have received much lessattention compared to*antemortem*MRI. Our study used a relativelylarge dataset of 135 high-resolution T2w 7 T postmortem whole-hemisphere MRI scansto evaluate multiple deep learning image segmentation architectures and to developan automatic segmentation pipeline that labels cortical gray matter, foursubcortical structures (caudate, globus pallidus, putamen, and thalamus), WMH, andnormal-appearing white matter. We report good agreement between thickness measuresderived from our deep learning pipeline with the reference standard ofsemi-automated thickness measurement. Our analysis linking morphometry measures andpathology demonstrated that automated analysis of postmortem MRI yields similarfindings to a labor-intensive semi-automated approach, and more broadly, thatautomated segmentation of postmortem MRI can complement and inform antemortemneuroimaging in neurodegenerative diseases. We have released our pipeline as astand-alone containerized tool that can be readily applied to other postmortem braindatasets.

## Supplementary Material

Supplementary Material

Supplementary Spreadsheet

## Data Availability

We have provided the code, scripts, and Jupyter notebooks to reproduce the findingsof this study at the project webpage. The MRI data will be available upon requestdue to compliance and ethical issues.
