# Supplementary material for: Automated deep learning segmentation of high-resolution 7 Tesla postmortem MRI for quantitative analysis of structure-pathology correlations in neurodegenerative diseases
Source: Imaging Neurosci (Camb). 2024 May 8;2:imag-2-00171. doi: 10.1162/imag_a_00171 (PMC11409836; doi:10.1162/imag_a_00171)
Supplement: Supplementary Material [file imag_a_00171-supp.pdf]

SUPPLEMENTARY

Automated deep learning segmentation of high-resolution 7 tesla postmortem MRI for quantitative analysis of structure-pathology correlations in neurodegenerative diseases

Pulkit Khandelwal[a,b,**], Michael Tran Duong[a], Shokufeh Sadaghiani[c], Sydney Lim[b,d], Amanda E. Denning[b,d], Eunice Chung[b,d], Sadhana Ravikumar[b,d], Sanaz Arezoumandan[c], Claire Peterson[c], Madigan Bedard[b,d], Noah Capp[c], Ranjit Ittyerah[b,d], Elyse Migdal[c], Grace Choi[c], Emily Kopp[c], Bridget Loja[c], Eusha Hasan[c], Jiacheng Li[c], Alejandra Bahena[c], Karthik Prabhakaran[d], Gabor Mizsei[d], Marianna Gabrielyan[c], Theresa Schuck[e], Winifred Trotman[c], John Robinson[e], Daniel T. Ohm[c], Edward B. Lee[e], John Q. Trojanowski[e,*], Corey McMillan[c], Murray Grossman[c,*], David J. Irwin[c], John A. Detre[c], M. Dylan Tisdall[d], Sandhitsu R. Das[b,c], Laura E.M. Wisse[f], David A. Wolk[c], Paul A. Yushkevich[b,d]

[a]*Department of Bioengineering, University of Pennsylvania, Philadelphia, USA*
[b]*Penn Image Computing and Science Laboratory, University of Pennsylvania, Philadelphia, USA*
[c]*Department of Neurology, University of Pennsylvania, Philadelphia, USA*
[d]*Department of Radiology, University of Pennsylvania, Philadelphia, USA*
[e]*Department of Pathology and Laboratory Medicine, University of Pennsylvania, Philadelphia, Pennsylvania, USA*
[f]*Department of Diagnostic Radiology, Lund University, Lund, Sweden*

Architectural details of the nine neural networks

3D Unet-like: We implement a custom in-house 3D Unet-like architecture Khandelwal and Yushkevich (2020), where the input and the output have dimensions *in*xHxWxD, and *out*xHxWxD. The first dimension represents the number of channels, and H, W and D denote the height, width and depth respectively. There are five encoder blocks (with the last block acting as the

*Author deceased.
**Corresponding author: Pulkit Khandelwal
Richards Medical Research Laboratories
6th Floor 6025, 3700 Hamilton Walk, Philadelphia, PA 19104
Email address: pulks@seas.upenn.edu

bottleneck layer), and four decoder blocks, with skip connections. We have used *groupnorm* as the normalization method. Note that the first encoder block does not consist of the MaxPool operation, and the first decoder block does not consist of the first set of conv3D, groupnorm, and ReLU units. The ***encoder*** block has the following *output* dimensions at each of the encoder block: E1: 16xHxWxD, E2: 32x$\frac{H}{2}$x$\frac{W}{2}$x$\frac{D}{2}$, E3: 64x$\frac{H}{4}$x$\frac{W}{4}$x$\frac{D}{4}$, E4: 128x$\frac{H}{8}$x$\frac{W}{8}$x$\frac{D}{8}$, E5: 256x$\frac{H}{16}$x$\frac{W}{16}$x$\frac{D}{16}$. The ***decoder*** block has the following *output* dimensions at each of the decoder block: D1: 256x$\frac{H}{8}$x$\frac{W}{8}$x$\frac{D}{8}$, D2: 128x$\frac{H}{4}$x$\frac{W}{4}$x$\frac{D}{4}$, D3: 64x$\frac{H}{2}$x$\frac{W}{2}$x$\frac{D}{2}$, D4: 32xHxWxD.

VNet: We implement the following variant of VNet Milletari et al. (2016). The input and the output volumes have dimensions *in*xHxWxD (here: *in* is 1), and *out*xHxWxD respectively, where *out* represents the number of output classes. We have four different modules: an InputTransition, DownTransition, UpTransition, and OutputTransition. The InputTransition comprises of a 3D convolution block with input channels *in* and produces *ch* output channels by using a kernel size of 5 and padding of 2, followed by BatchNorm. Next, the input is added to the output of the convolutional block to obtain a residual function and then followed by PReLU, to produce an output with dimensions, *ch*xHxWxD. This output is then fed to the first DownTransition module. The DownTransition block consists of 3D convolution block, with kernel size of 2 and stride of 2, which takes in the input *ch*xHxWxD and downsamples the feature map by a factor of 2 and doubles the number of channels, thus giving an output of ($2\times ch$)x$\frac{H}{2}$x$\frac{W}{2}$x$\frac{D}{2}$, which is then followed by BatchNorm and PReLU nonlinearity and an optional dropout. A residual function is then created by using another convolutional block by using a kernel size of 5 and padding of 2, followed by PReLU nonlinearity. The second DownTransition block produces an output of ($4\times ch$)x$\frac{H}{4}$x$\frac{W}{4}$x$\frac{D}{4}$. The third and fourth DownTransition blocks has the dropout set to True and produces an output of ($8\times ch$)x$\frac{H}{8}$x$\frac{W}{8}$x$\frac{D}{8}$ and ($16\times ch$)x$\frac{H}{16}$x$\frac{W}{16}$x$\frac{D}{16}$ respectively. The UpTransition block is similar to the DownTransition with the difference that every block upsamples the input using 3D transpose convolutions, and has skip connections like UNet from the corre-

sponding DownTransition block. The first UpTransition block upsamples from $(16\times ch)$x$\frac{H}{16}$x$\frac{W}{16}$x$\frac{D}{16}$ to $(16\times ch)$x$\frac{H}{8}$x$\frac{W}{8}$x$\frac{D}{8}$ with dropout set to true. The next UpTransition block upsamples to $(8\times ch)$x$\frac{H}{4}$x$\frac{W}{4}$x$\frac{D}{4}$ with dropout set to true. The next two UpTransition blocks produces an output of $(4\times ch)$x$\frac{H}{2}$x$\frac{W}{2}$x$\frac{D}{2}$ and $(2\times ch)$xHxWxD respectively. Finally, the last block OutputTransition produces the desired output of *out*xHxWxD using two 3D convolutional layers.

VoxResNet: We implement the following variant of VoxResNet Chen et al. (2018). In our implementation, we define a building block called VoxRes, which creates a residual function, comprising two sets of BatchNorm, ReLU and 3D convolution with kernel size of 3, stride of 1 and padding of 1. In the VoxResNet architecture, there are four encoder and four decoder blocks. The first encoder block consists of a 3D convolution with kernel size 3 and padding of 1, BatchNorm, ReLU and 3D convolution, thus changing the input of *in*xHxWxD to *ch*xHxWxD, where ch=32. The next three encoder block successively downsamples the input from *ch*xHxWxD to $(2\times ch)$x$\frac{H}{2}$x$\frac{W}{2}$x$\frac{D}{2}$, $(2\times ch)$x$\frac{H}{4}$x$\frac{W}{4}$x$\frac{D}{4}$ and $(2\times ch)$x$\frac{H}{8}$x$\frac{W}{8}$x$\frac{D}{8}$ respectively by stacking BatchNorm, ReLU, 3D convolution, with kernel size of 3, stride of (1, 2, 2) and padding o0f 1, and VoxResNet module. The four decoder blocks takes in the output of the corresponding encoder block. The decoder block transforms the output of the first encoder from *ch*xHxWxD to *ch*xHxWxD using transposed convolution and then from *ch*xHxWxD to *out*xHxWxD using convolution where *out* represents the number of output classes. The second decoder block takes in output from the corresponding encoder block to produce an output from $(2\times ch)$x$\frac{H}{2}$x$\frac{W}{2}$x$\frac{D}{2}$ to *out*xHxWxD using transposed convolution with 64 filters and a 3D convolution with kernel of (1, 2, 2) and stride of (1, 2, 2). Similarly, the third and fourth decoder block takes in output from the corresponding encoder block to produce an output from $(2\times ch)$x$\frac{H}{4}$x$\frac{W}{4}$x$\frac{D}{4}$ to *out*xHxWxD using transposed convolution with 64 filters with kernel of (1, 4, 4) and stride of (1, 4, 4) and a 3D convolution; and from $(2\times ch)$x$\frac{H}{8}$x$\frac{W}{8}$x$\frac{D}{8}$ to *out*xHxWxD using transposed convolution with 64 filters with kernel of (1, 8, 8) and stride of (1, 8, 8) and a 3D convolution. The outputs from the four decoder blocks are then added element-wise to

produce the final output of size outxHxWxD.

Attention U-Net: The building block of Attention U-Net Oktay et al. (2018) is the AttentionGate module which comprises of a gating mechanism to suppress irrelevant background. The AttentionGate takes in two volumes G and X, of size ch_gxH_gxW_gxD_g and ch_xxH_xxW_xxD_x respectively, and produce outputs of size ch_{inter}xH_gxW_gxD_g using 3D convolutions with kernel size and padding of 1. The two outputs are then summed up element-wise and passed through ReLU activation, followed by another convolution, with kernel size and padding of 1, and a sigmoid activation function. Finally, this output is upsampled and then multiplied by the input volume X to ch_xxH_xxW_xxD_x to produce the final volume, $\hat{X}$, with dimensions ch_xxH_xxW_xxD_x.

The Attention U-Net architecture mimics a standard U-Net with an AttentionGate mechanism taking in input from the decoder's output for upsampling and the corresponding encoder. The four encoder blocks take in the volume of inxHxWxD, where in is 1, and successively reduce the resolution to $(64\times ch)$x$\frac{H}{2}$x$\frac{W}{2}$x$\frac{D}{2}$, $(128\times ch)$x$\frac{H}{4}$x$\frac{W}{4}$x$\frac{D}{4}$, $(256\times ch)$x$\frac{H}{8}$x$\frac{W}{8}$x$\frac{D}{8}$, and $(512\times ch)$x$\frac{H}{16}$x$\frac{W}{16}$x$\frac{D}{16}$ by using convolution block comprising of a stack of two convolutions with kernel size of (2,2,2) and two ReLU layers.

Now, the first AttentionGate takes in as inputs, the outputs of the third and the fourth encoder blocks, and produces an output of dimension $(256\times ch)$x$\frac{H}{8}$x$\frac{W}{8}$x$\frac{D}{8}$, which is then concatenated with the upsampled version of the output of the fourth encoder block $(256\times ch)$x$\frac{H}{8}$x$\frac{W}{8}$x$\frac{D}{8}$, and then this output is again upsampled to $(128\times ch)$x$\frac{H}{8}$x$\frac{W}{8}$x$\frac{D}{8}$. This procedure is repeated two more times to get output dimensions of $(64\times ch)$x$\frac{H}{4}$x$\frac{W}{4}$x$\frac{D}{4}$ and $(64\times ch)$x$\frac{H}{2}$x$\frac{W}{2}$x$\frac{D}{2}$ respectively. A final upsampling block then produces the desired output of outxHxWxD.

AnatomyNet (Vanilla): AnatomyNet Zhu et al. (2019) is a variant of U-Net which takes advantage of the Squeeze-and-Excitation residual feature (SE) blocks proposed in Rickmann et al. (2019); Roy et al. (2018) to segment small anatomical structures, which are often missed by networks such as U-Net. We implement four variants of the AnatomyNet, with the first variant

AnatomyNet (Vanilla) without any SE blocks. The first block takes an input with size *in*xHxWxD and generates a feature map of size *ch*xHxWxD, here *in* is 1 and *ch* is 28, using 3D convolution with kernel size of 3, stride of 2, and padding of 1; followed by a BatchNorm and LeakyReLU activation layer.

The next three blocks take in the input *ch*xHxWxD, and successively increase the feature channels and downsamples by 2 from (28)xHxWxD to (34)x$\frac{H}{2}$x$\frac{W}{2}$x$\frac{D}{2}$. Each of these three blocks consists of two repetitions of a ResidualBasicBlock. The ResidualBasicBlock comprises of a 3D convolution with kernel size of 3, stride of 2, and padding of 1, followed by BatchNorm, LeakyReLU, 3D convolution with kernel size of 3, stride of 2, and padding of 1, BatchNorm, SEBlock and a downsampling 3D convolution with kernel size of 3, stride of 1. The SEBlock consists of one of the three variants of the Squeeze-and-Excitation blocks: Spatial Excitation AnatomyNet (SE), Channel Excitation AnatomyNet (CE) or the Channel-spatial Excitation AnatomyNet (CE + SE). But, here we do not include the SEBlock in the ResidualBasicBlock in the AnatomyNet (Vanilla) variant.

The next block in the network architecture is the ResidualUPBasicBlock, which concatenates two inputs of size (34)x$\frac{H}{2}$x$\frac{W}{2}$x$\frac{D}{2}$ and (32)x$\frac{H}{2}$x$\frac{W}{2}$x$\frac{D}{2}$ to produce (66)x$\frac{H}{2}$x$\frac{W}{2}$x$\frac{D}{2}$, followed by a 3D convolution with kernel size of 3, stride of 2, and padding of 1, followed by BatchNorm, LeakyReLU, 3D convolution with kernel size of 3, stride of 2, and padding of 1, BatchNorm, SEBlock and a downsampling 3D convolution with kernel size of 3 and stride of 1, and LeakyReLU to produce (32)x$\frac{H}{2}$x$\frac{W}{2}$x$\frac{D}{2}$. This is followed by three sets of ResidualBasicBlock, without the downsampling layer, and the ResidualUPBasicBlock to produce an output of size (14)xHxWxD. A final 3D convolution is employed to produce the output of (out)xHxWxD, where *out* is the number of segmentation classes.

Channel Squeeze and Spatial Excitation AnatomyNet (sSE): The architecture of the Channel Squeeze and Spatial Excitation AnatomyNet (sSE) is similar to the *Vanilla* variant, with the only difference being that the SEBlock consists of Spatial excitation module. The SEBlock squeezes the feature maps along the channel dimension and excites the spatial dimension. This block helps

in segmentation of fine-grained structures as proposed in Roy et al. (2018). This block consists of a 3D convolution with kernel size of 1 and stride of 1, followed by a Sigmoid activation layer which takes an input with ch channels, chxHxWxD, and reduces it to 1xHxWxD. The channel-squeezed output is then multiplied element-wise with the input feature map to produce the spatial-excited feature map of dimension chxHxWxD.

Spatial Squeeze and Channel Excitation AnatomyNet (cSE): The architecture of the Spatial Squeeze and Channel Excitation AnatomyNet (cSE) is similar to the *Vanilla* variant, with the only difference being that the SEBlock consists of a Channel excitation module. This SEBlock consists of the following layers: the spatial channels of dimension chxHxWxD, which is spatially squeezed to chx1x1x1 using adaptive pooling. Two layers of fully connected layer and ReLU activation function transforms this feature map to a vector of length ch. This feature map is then element-wise multiplied with the original input feature map to produce a channel-excited map of chxHxWxD.

Spatial and Channel Squeeze and Excitation AnatomyNet (scSE): The architecture of the Spatial excitation AnatomyNet (SE) is similar to the *Vanilla* variant, with the only difference being that the SEBlock consists of Channel-spatial excitation module. The input tensor of size chxHxWxD is passed through both cSE and sSE. An element-wise max is taken between these feature maps to produce the final output of size chxHxWxD.

nnU-Net: We use the default network in the nnU-Net framework as described in Isensee et al Isensee et al. (2021).

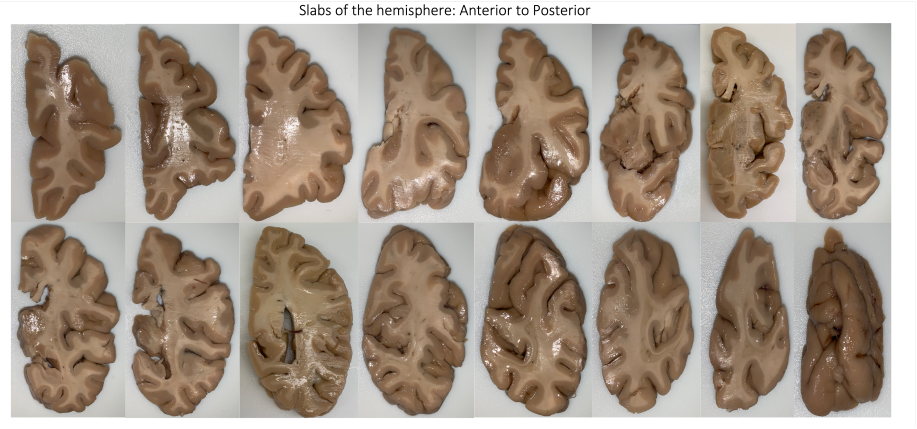

Figure 1: Postmortem tissue blockface photograph of a patient with Parkinson's disease (not demented) and Lewy body disease, deceased at the age of 79. Shown are the slabbed blockface images from anterior to posterior.

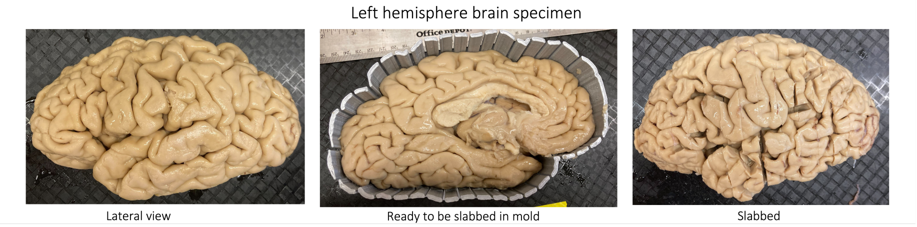

Figure 2: Post-mortem tissue blockface photograph of a patient with FTLD primary-progressive-aphasia (PNFA) and globular glial tauopathy (GGT) disease, deceased at the age of 74. Shown are the lateral and medial views of the left hemisphere. The tissue is then placed in a mold and is subsequently slabbed.

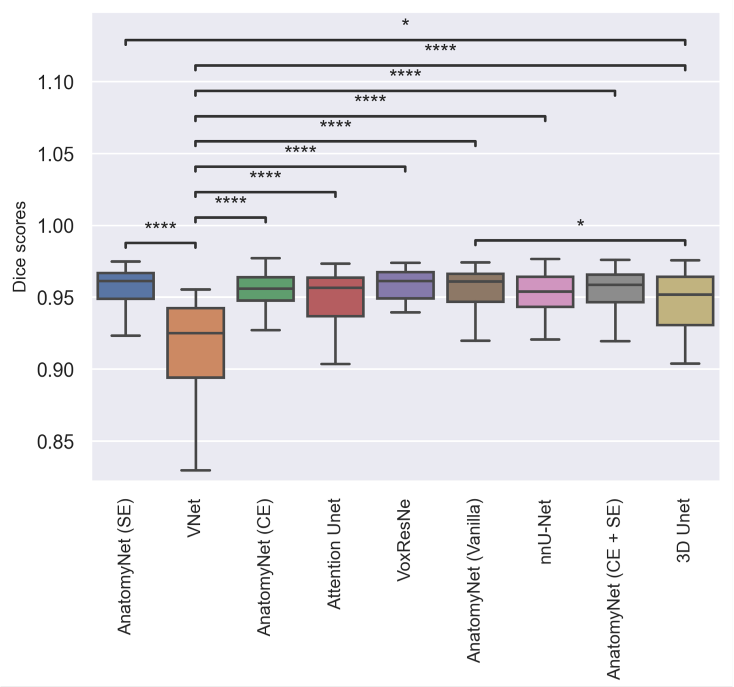

(a)

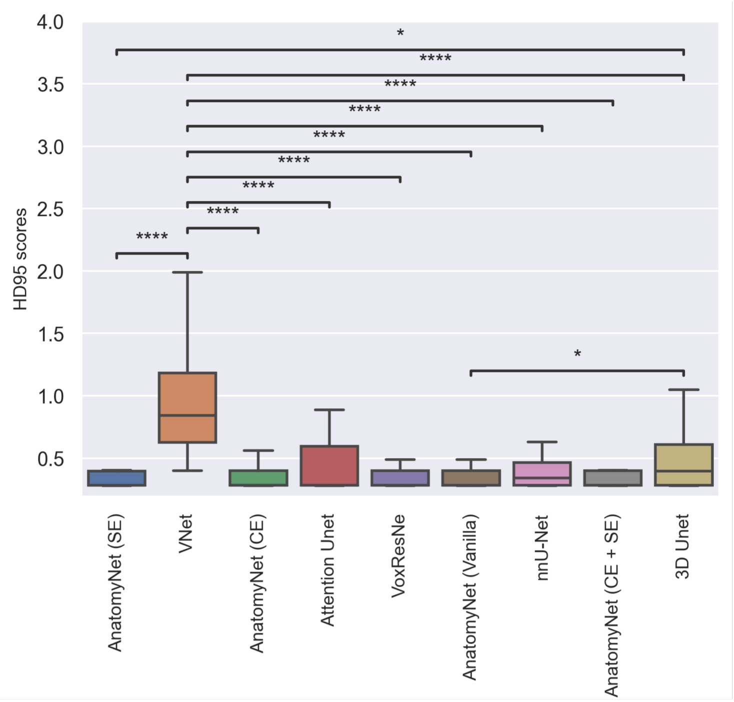

(b)

Figure 3: Shown are the Dice and the HD95 scores as box plots with pairwise paired t-tests after Bonferroni correction for all the nine network architectures in Table 2 of the main paper. Legend: *: $1.00 \times 10^{-2} < p \leq 5.00 \times 10^{-2}$; **: $1.00 \times 10^{-3} < p \leq 1.00 \times 10^{-2}$; ***: $1.00 \times 10^{-4} < p \leq 1.00 \times 10^{-3}$; ****: $p \leq 1.00 \times 10^{-4}$.

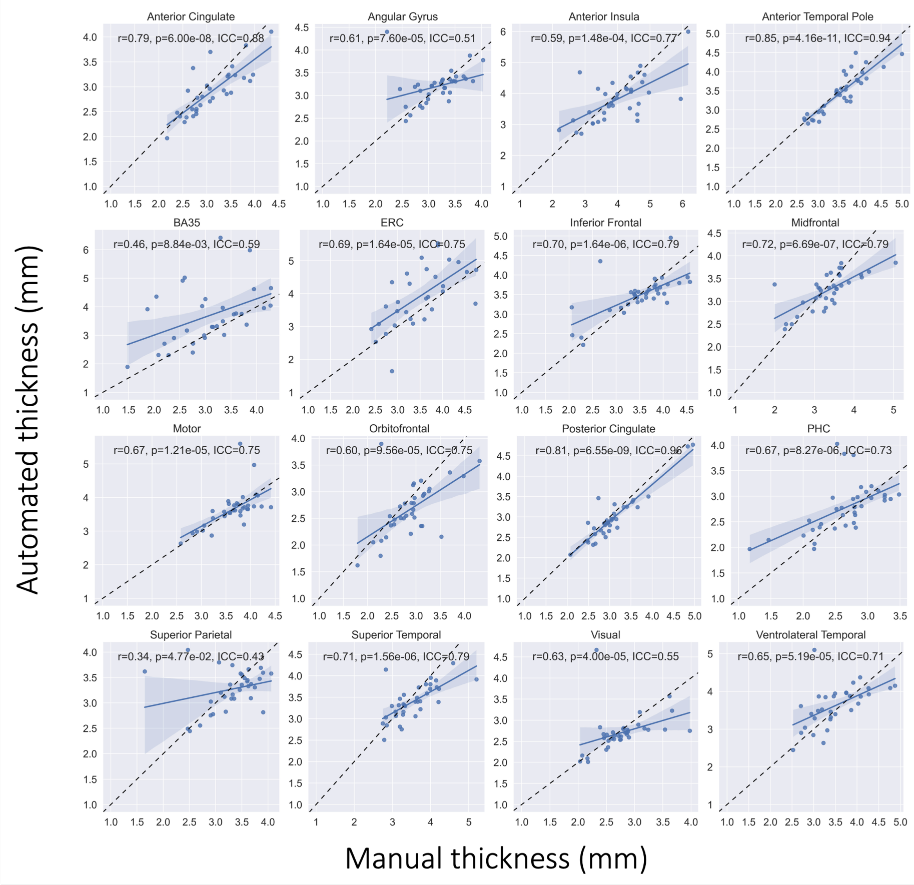

Figure 4: Regional cortical thickness measurements. Figure shows correlation plots between cortical thickness measured by the automated nnU-Net-CRUISE (y-axis) and reference standard manual segmentations (x-axis). Within each plot, we tabulate Spearman's correlation coefficient (r), p value, and the Average fixed raters Intra-class correlation coefficient (ICC) scores. The dashed line indicates $y = x$.

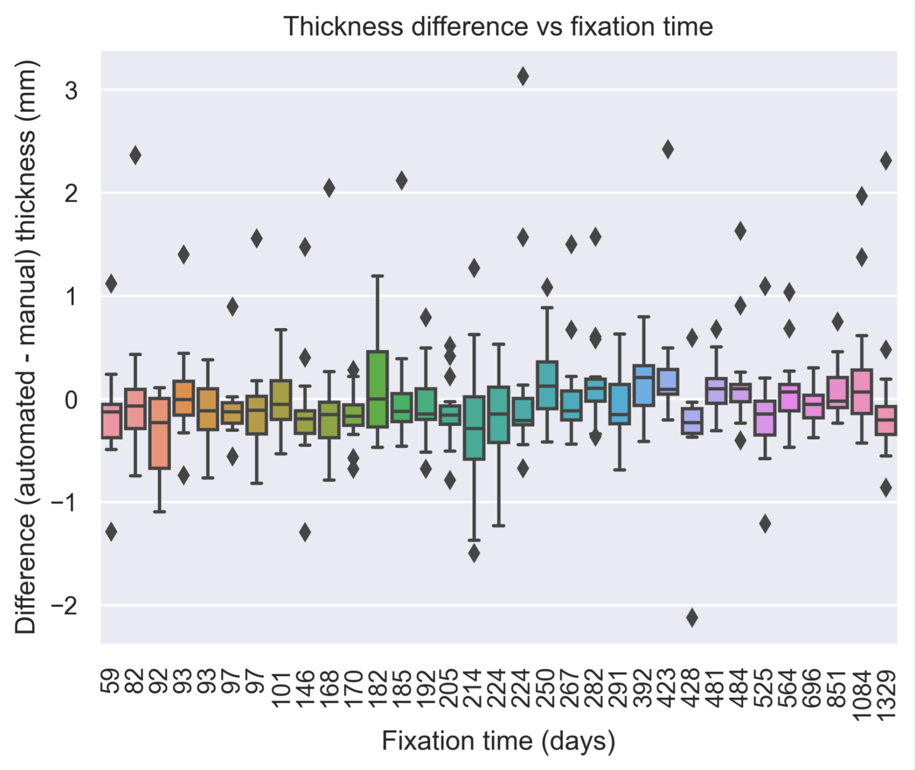

Figure 5: The plot shows the mismatch in thickness (in mm) between automated (nnU-Net-CRUISE) and manual reference segmentations vs fixation time (in days). Each box plot represents the distribution of a subject's differences in thickness values for all the 16 cortical regions. The plot does not reveal a systematic relationship between fixation time and thickness mismatch, neither in terms of bias nor in terms of variance. This is consistent with the literature that shows relative plateauing of T2 values in postmortem MRI after initial 1-2 months fixation Dawe et al. (2009). We conclude that the large variation in fixation times in our study did not significantly affect the thickness computation based on the obtained segmentations.

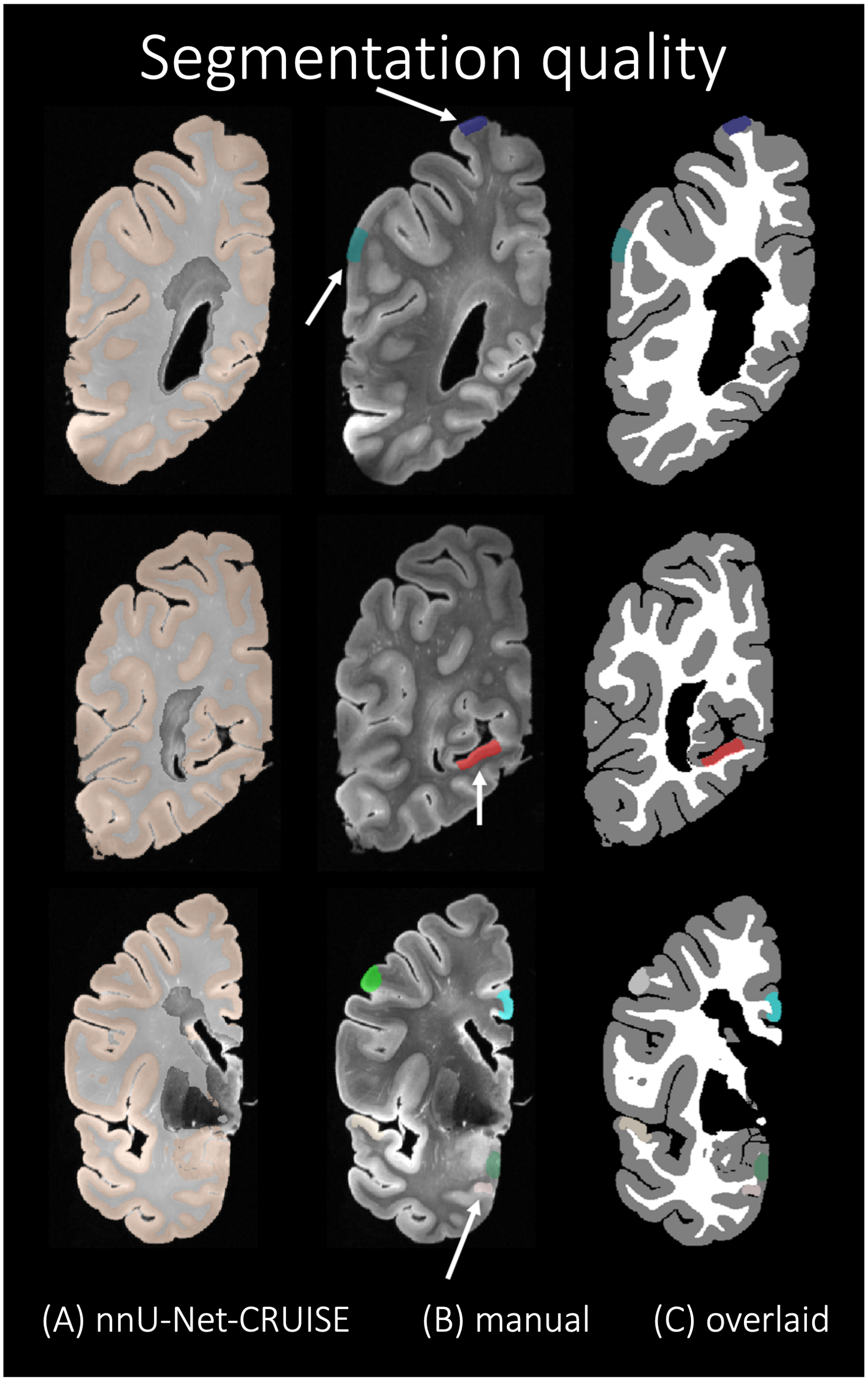

Figure 6: Segmentation quality. We show a sample segmentation where we observe some discrepancy between the manual reference and automated nnU-Net-CRUISE segmentations. For a single subject in the coronal view: (A) nnU-Net-CRUISE segmentation overlaid on MRI. (B) manual reference segmentations marked with arrows for the four cortical regions of interest: visual (red), superior parietal (blue), angular gyrus (cyan), BA35 (cream) overlaid on the MRI. (C) manual reference overlaid on nnU-Net-CRUISE segmentations. We observe that the superior parietal is under-segmented by the manual rater and visual is over-segmented by the rater.

Table 1: Brief demographic summary of the subjects used for training and evaluation of the deep learning models in the current study. Abbreviations: AD: Alzheimer's Disease, ALS: amyotrophic lateral sclerosis, CVD: cerebrovascular disease, LATE: limbic-predominant age-related TDP-tau 43 encephalopathy, LBD: Lewy body disease, CTE: chronic traumatic encephalopathy, FTLD-TDP: frontotemporal lobar degeneration with TDP inclusions, GGT: globular glial p-tauopathy, CVD: cerebrovascular disease, CBD: corticobasal degeneration, PART: Primary age-related p-tauopathy, PSP: progressive supranuclear palsy, p-tau-Misc: p-tauopathy unclassifiable.

Subject	AD Spectrum	Primary	Secondary	Age	Race	Sex	Cortex cross-val	Subcortical cross-val	WMH cross-val	WM	PMI	Fixation time (days)
2	Yes	AD	LATE+AD	87	White	M			Y		14	NA
4	Yes	AD	LATE+AD	89	White	F		Y		Y	19	481
9	Yes	AD	LBD+LATE+AD	63	White	F			Y		20	696
13	Yes	AD	AD	73	White	M	Y	Y	Y		7	512
17	Yes	LBD	LBD+AD+CTE	71	White	M	Y	Y	Y		21	59
20	Yes	LBD	LBD+AD	73	Black	M	Y	Y	Y		17	224
26	No	FTLD-TDP	FTLD-TDP43	65	White	M	Y	Y	Y		11	93
29	No	FTLD-TDP	FTLD+AD	81	White	M	Y	Y			14	267
31	No	Tau-Misc	FTLD	50	White	M			Y		18	185
35	No	Tau-4R	CBD	77	White	F	Y	Y		Y	21	1084
37	No	Tau-4R	CBD+AD	76	White	M			Y		12	250
38	Yes	AD	LATE+AD	83	White	F			Y		8	525

Table 2: The locations from where the neuropathology ratings were obtained from, either the exact (main regions) or the closest (exploratory regions) to the cortical brain regions.

Brain region	Matching pathology region	Exact
Visual cortex	Occipital cortex	Y
Middle frontal gyrus	Middle frontal gyrus	Y
Orbitofrontal gyrus	Orbital frontal cortex	Y
Anterior cingulate	Cingulate gyrus	Y
Posterior cingulate	Cingulate gyrus	Y
Motor cortex	Motor cortex	Y
Angular gyrus	Angular gyrus	Y
Superior parietal cortex	Angular gyrus	N
Superior temporal cortex	Superior/ middle temporal	Y
Anterior temporal pole	Amygdala	N
Anterior insula	Middle frontal gyrus	N
Ventrolateral part of anterior temporal cortex (Inferior temporal gyrus)	Entorhinal cortex	N
Inferior frontal cortex (Broca's area)	Middle frontal gyrus	N
Entorhinal cortex	Entorhinal cortex	Y
Brodmann area 35	Entorhinal cortex	Y
Parahippocampal cortex	CA1/ Subiculum	N

Table 3: Associations between morphometric measures and summary measures of tau pathology and neuronal loss in the medial temporal lobe, a region linked to early neurodegeneration in Alzheimer's disease. Shown is the one-sided Spearman's correlation (controlling for age, sex and PMI) between the cortical thickness measures derived from the topologically corrected nnU-Net-CRUISE gray matter segmentation with corresponding MTL ratings of p-tau pathology and neuronal loss density. Each cell is color coded with darker shades indicating more negative correlations. CI indicates 95% confidence interval. The asterisk indicates that the test survived Bonferroni multiple testing correction. Legend *:$0.01 < p \leq 0.05$; **:$0.001 < p \leq 0.01$; ***:$0.0001 < p \leq 0.001$; ****:$0.00001 < p \leq 0.0001$.

Pathology ratings	p-tau (MTL)		Neuronal Loss (MTL)	
**ROI**	**rho**	**CI**	**rho**	**CI**
**Visual**	-0.1	[-1.0, 0.09]	-0.105	[-1.0, 0.08]
**Motor cortex**	-	-	-	-
**Posterior cingulate**	-0.337	[-1.0, -0.13]	-0.396 ***	[-1.0, -0.2]
**Midfrontal**	-0.202	[-1.0, -0.01]	-0.177	[-1.0, 0.01]
**Anterior cingulate**	-0.215	[-1.0, -0.01]	0.012	[-1.0, 0.22]
**Orbitofrontal**	-	-	-	-
**Superior temporal pole**	-0.115	[-1.0, 0.08]	-0.153	[-1.0, 0.04]
**Inferior frontal**	-0.027	[-1.0, 0.16]	-0.033	[-1.0, 0.15]
**Anterior insula**	-0.228	[-1.0, -0.04]	-0.213	[-1.0, -0.03]
**Anterior temporal pole**	-0.226	[-1.0, -0.04]	-0.204	[-1.0, -0.02]
**Ventrolateral temporal cortex**	-0.14	[-1.0, 0.09]	-0.049	[-1.0, 0.18]
**Superior parietal**	-0.194	[-1.0, 0.0]	-0.063	[-1.0, 0.14]
**Angular gyrus**	-0.285	[-1.0, -0.1]	-0.135	[-1.0, 0.05]
**Entorhinal cortex**	-0.53 ****	[-1.0, -0.38]	-0.47 ****	[-1.0, -0.31]
**Brodmann Area 35**	-0.18	[-1.0, 0.01]	-0.321 **	[-1.0, -0.14]
**Parahippocampal cortex**	-0.19	[-1.0, -0.0]	-0.195	[-1.0, -0.01]

Table 4: Morphometry associations with underlying Neuropathology. Shown is the one-sided Spearman's correlation (controlling for age, sex and PMI) between regional cortical thickness measures derived from topologically corrected nnU-Net-CRUISE gray matter segmentation with corresponding regional and the medial temporal lobe (MTL) ratings of p-tau pathology, neuronal loss density, and global amyloid-β ratings, CERAD score, and Braak staging. The analyses is repeated for thickness measurements based-on manual segmentations. Each cell is color coded with darker shades indicating more negative correlations. The asterisk indicates that the test survived Bonferroni multiple testing correction Bonferroni (1935).

Pathology ratings	Abeta		Braak		CERAD		p-tau		Neuronal loss		p-tau (MTL)		Neuronal loss (MTL)	
ROI	nnU-Net-CRUISE	Manual	nnU-Net-CRUISE	Manual	nnU-Net-CRUISE	Manual	nnU-Net-CRUISE	Manual	nnU-Net-CRUISE	Manual	nnU-Net-CRUISE	Manual	nnU-Net-CRUISE	Manual
VIS	0.049	0.054	-0.111	-0.164	0.033	-0.104	-0.055	-0.194	0.04	-0.026	-0.085	-0.049	-0.259	-0.243
MOT	-0.138	0.095	-0.038	0.137	0.063	-0.09	-	-	-	-	-	-	-	-
PCIN	-0.443	-0.508	-0.449	-0.571	-0.465	-0.544	-0.439	-0.442	-0.499	-0.549	-0.489	-0.536	-0.473	-0.541
MF	-0.508	-0.297	-0.364	-0.33	-0.143	-0.142	-0.479	-0.363	-0.463	-0.528	-0.323	-0.192	-0.093	-0.058
ACIN	0.037	-0.185	0.017	-0.087	-0.1	-0.08	0.078	0.125	-0.149	-0.057	0.313	0.105	0.382	0.302
ORF	0.103	0.151	0.238	0.183	0.219	-0.115	-	-	-	-	-	-	-	-
STEMP	-0.028	0	0.076	-0.014	0.092	0.021	0.01	-0.045	0.196	0.146	0.098	0.096	0.212	0.152
IF	0.209	0.275	0.208	0.296	0.055	0.27	-0.026	0.073	0.016	0.024	0.323	0.374	0.327	0.422
ANTIN	-0.366	-0.381	-0.166	-0.39	-0.181	-0.457	-0.247	-0.434	-0.242	-0.455	-0.28	-0.398	-0.178	-0.268
ATEMPP	-0.568	-0.516	-0.649 *	-0.515	-0.435	-0.321	-0.615 *	-0.546	-0.461	-0.432	-0.512	-0.55	-0.363	-0.246
VLT	0.029	-0.264	0.21	-0.179	0.097	-0.183	0.047	-0.061	0.071	-0.26	0.179	-0.107	0.094	-0.15
SP	-0.207	-0.115	-0.13	-0.123	-0.195	-0.074	-0.275	-0.482	-0.153	-0.232	-0.208	-0.128	-0.148	-0.146
ANG	-0.33	-0.506	-0.42	-0.519	-0.205	-0.348	-0.485	-0.573 *	-0.46	-0.614 *	-0.31	-0.406	-0.378	-0.368
ERC	-0.256	-0.416	-0.247	-0.403	0.051	-0.289	-0.179	-0.231	-0.37	-0.395	-0.281	-0.453	-0.344	-0.407
BA35	-0.092	-0.29	-0.059	-0.424	-0.312	-0.303	-0.254	-0.388	-0.324	-0.365	-0.3	-0.485	-0.359	-0.373
PHC	-0.11	-0.374	-0.121	-0.543	0.009	-0.211	-0.076	-0.188	-0.1	-0.228	-0.125	-0.336	-0.085	-0.304

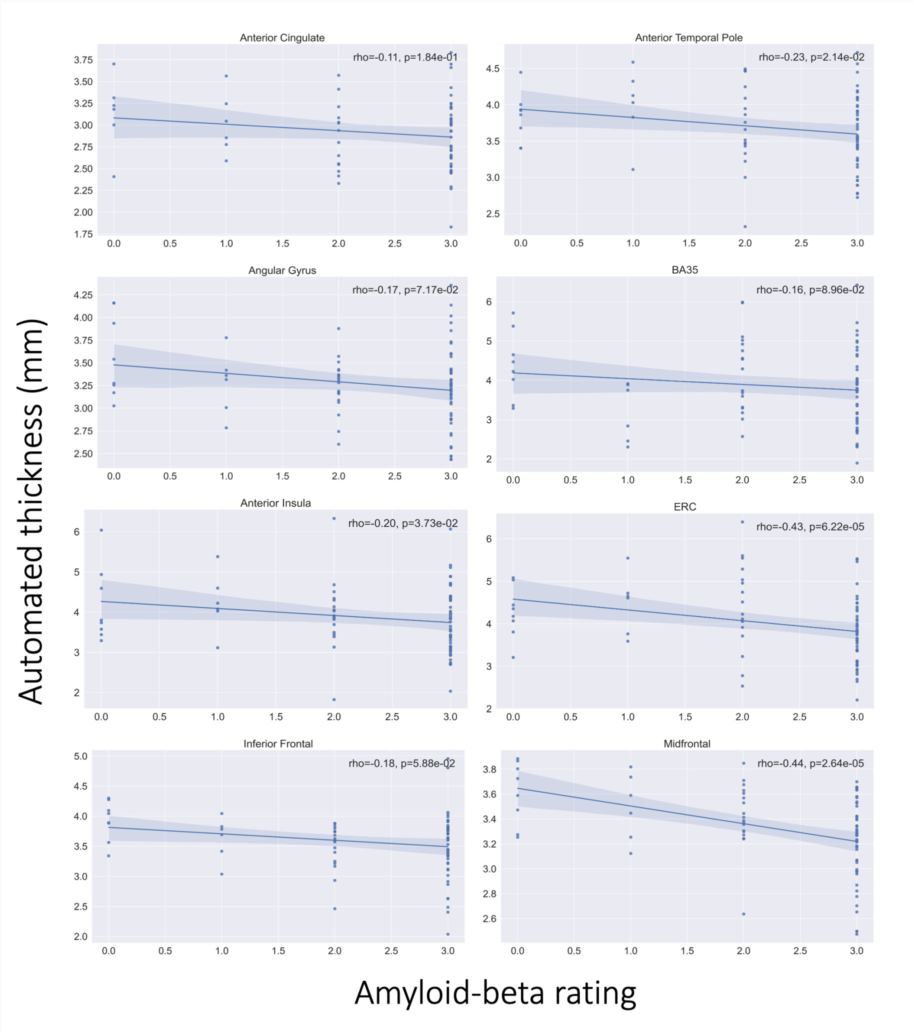

Figure 7: Spearman's correlation between cortical thickness measures derived from topologically corrected nnU-Net-CRUISE gray matter segmentation and global amyloid-β rating with p-value.

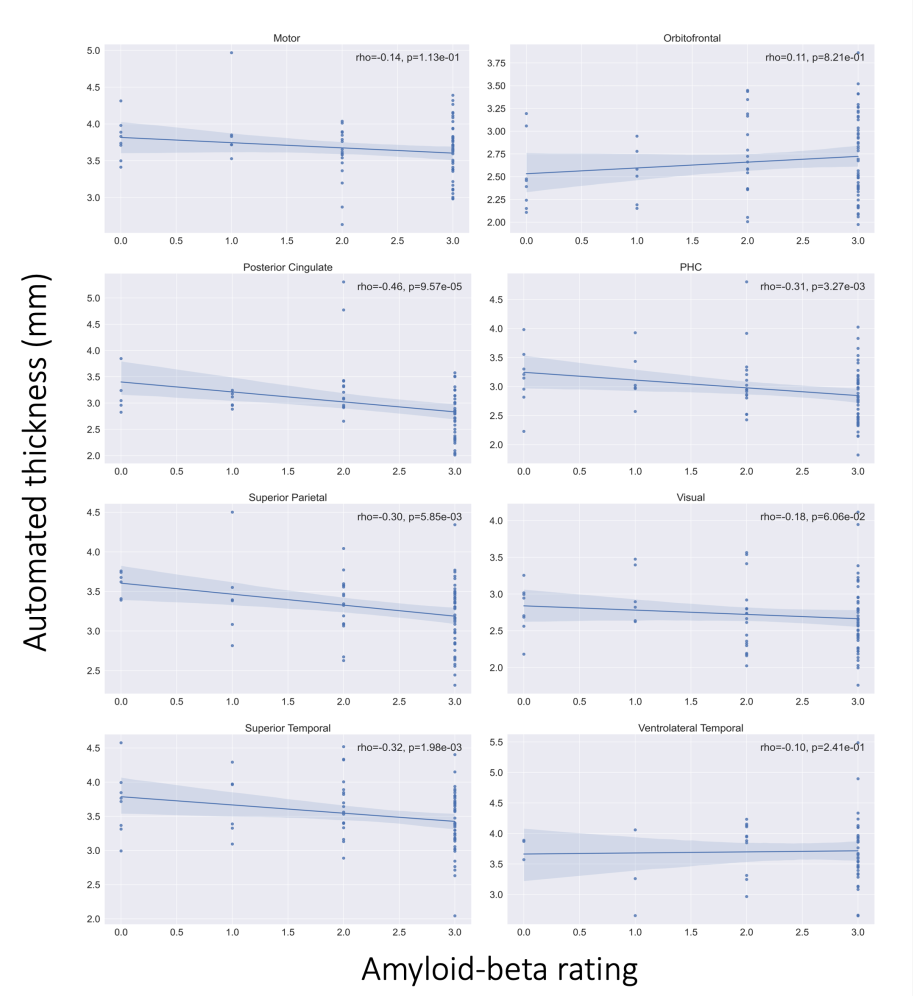

Figure 7: ***Continued.*** Spearman's correlation between cortical thickness measures derived from topologically corrected nnU-Net-CRUISE gray matter segmentation and global amyloid-β rating with p-value.

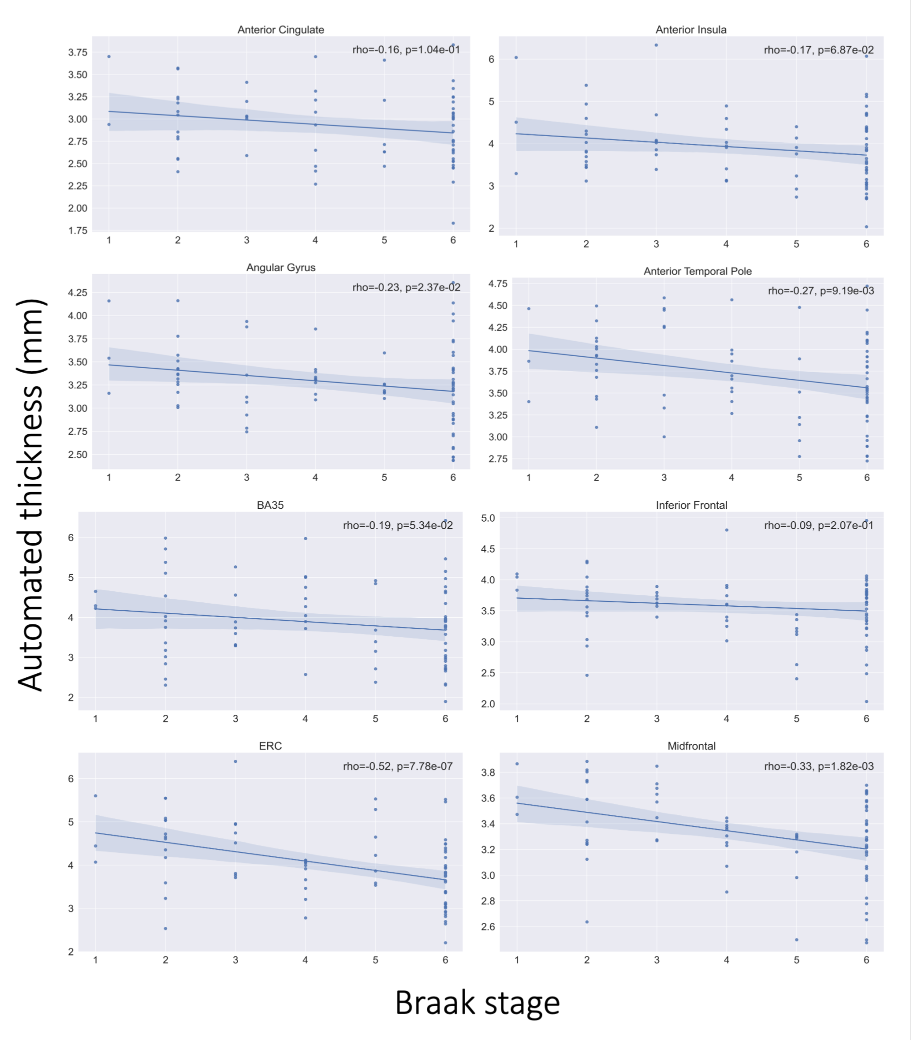

Figure 8: Spearman's correlation between cortical thickness measures derived from topologically corrected nnU-Net-CRUISE gray matter segmentation and Braak stage with p-value.

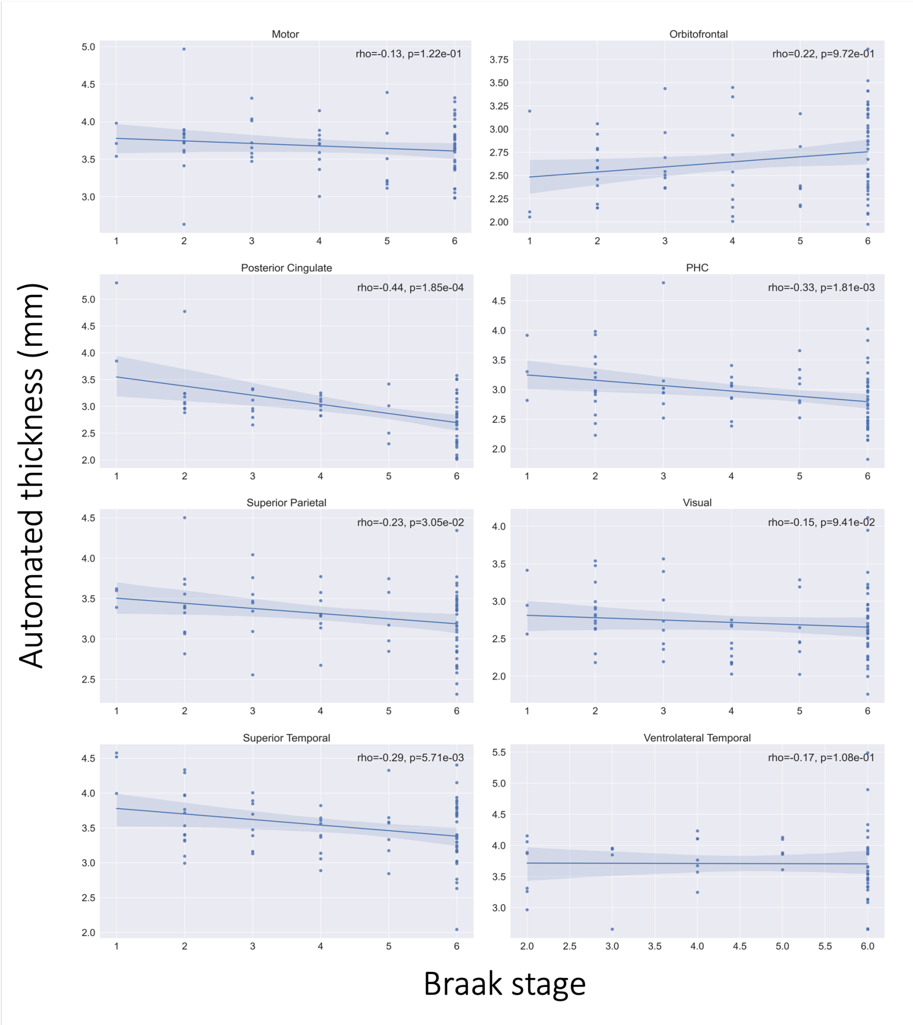

Figure 8: ***Continued.*** Spearman's correlation between cortical thickness measures derived from topologically corrected nnU-Net-CRUISE gray matter segmentation and Braak stage with p-value.

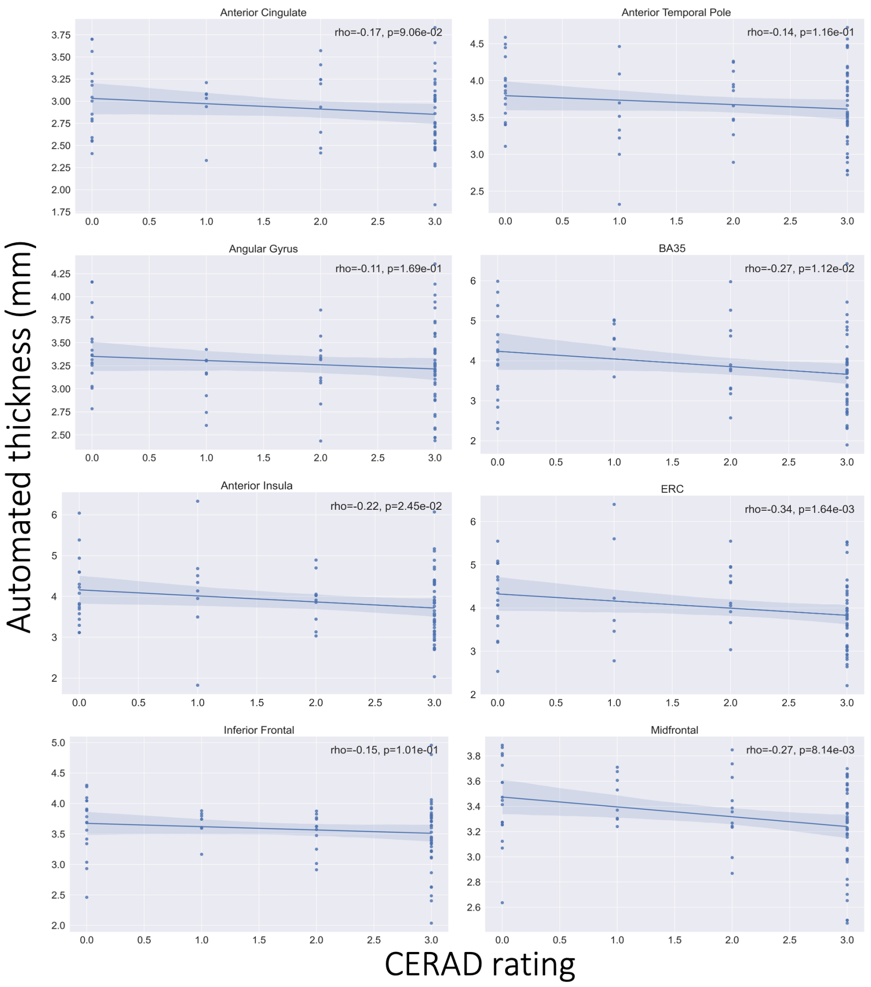

Figure 9: Spearman's correlation between cortical thickness measures derived from topologically corrected nnU-Net-CRUISE gray matter segmentation and global CERAD rating with p-value.

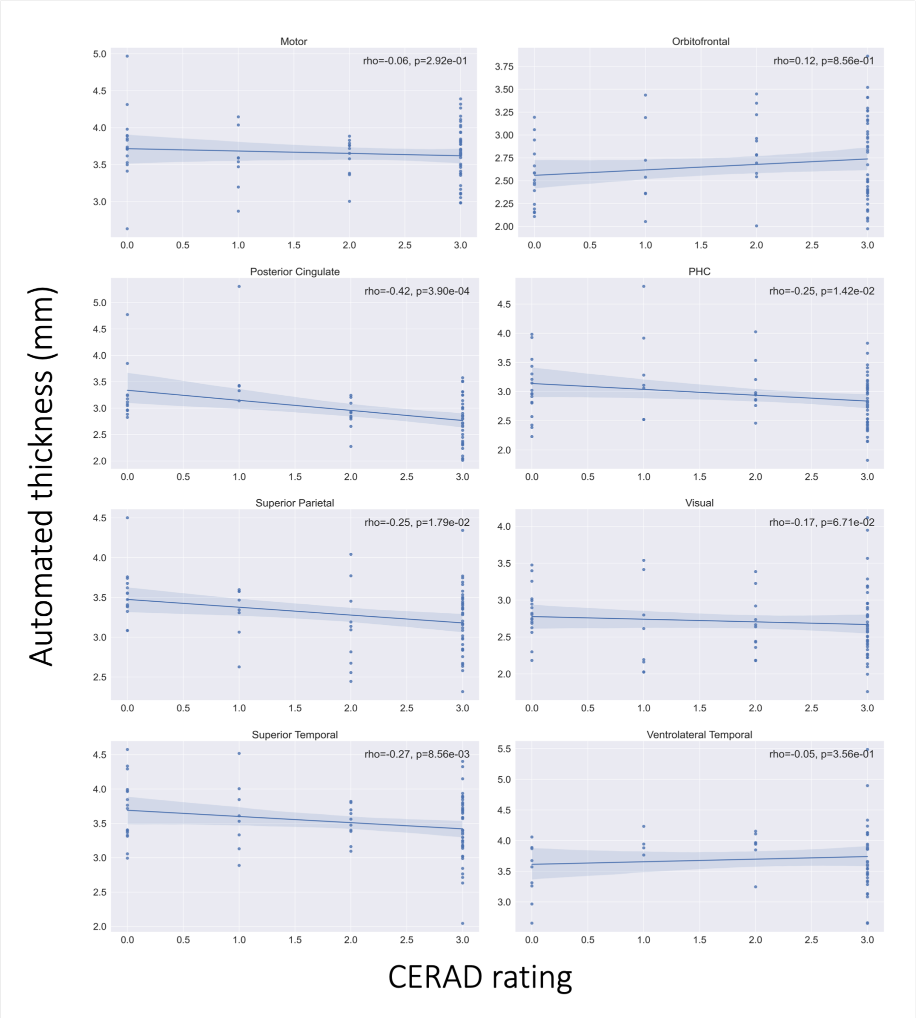

Figure 9: ***Continued.*** Spearman's correlation between cortical thickness measures derived from topologically corrected nnU-Net-CRUISE gray matter segmentation and global CERAD rating with p-value.

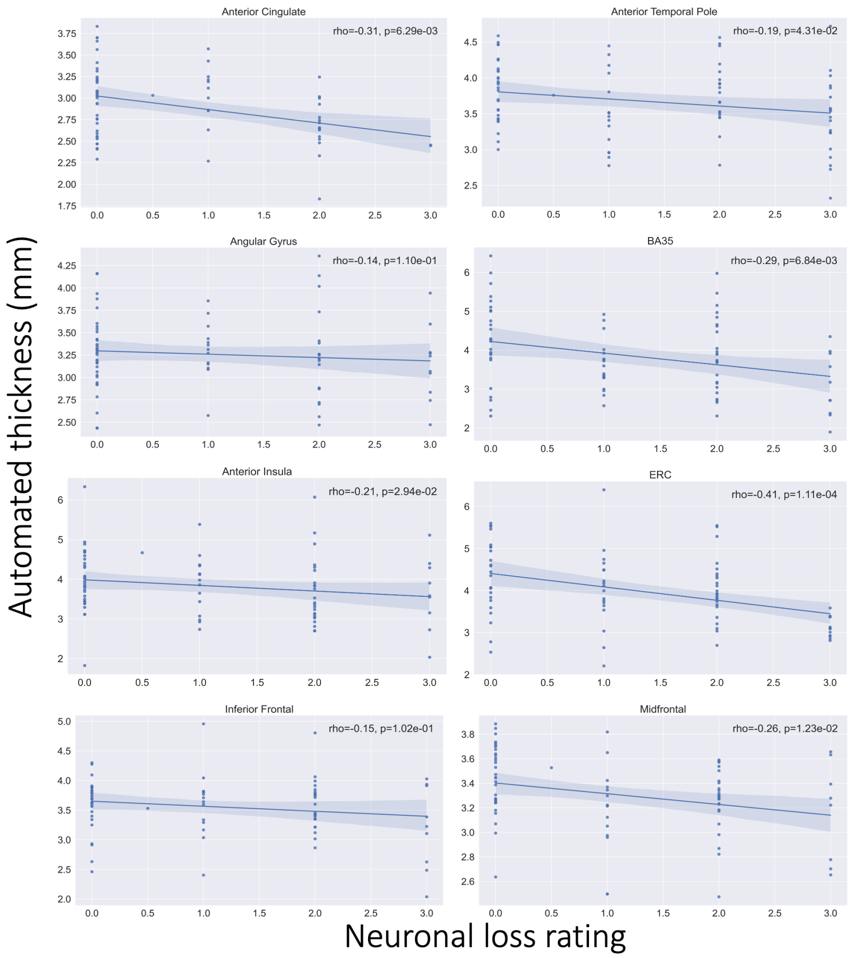

Figure 10: Spearman's correlation between cortical thickness measures derived from topologically corrected nnU-Net-CRUISE gray matter segmentation and regional neuronal loss rating with p-value.

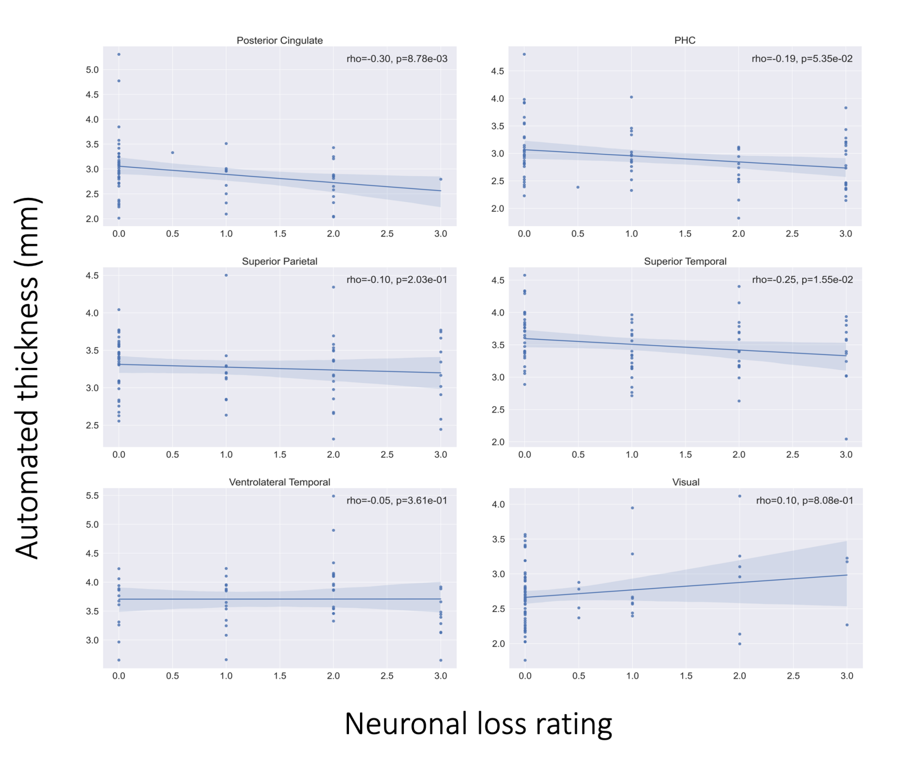

Figure 10: ***Continued.*** Spearman's correlation between cortical thickness measures derived from topologically corrected nnU-Net-CRUISE gray matter segmentation and regional neuronal loss rating with p-value.

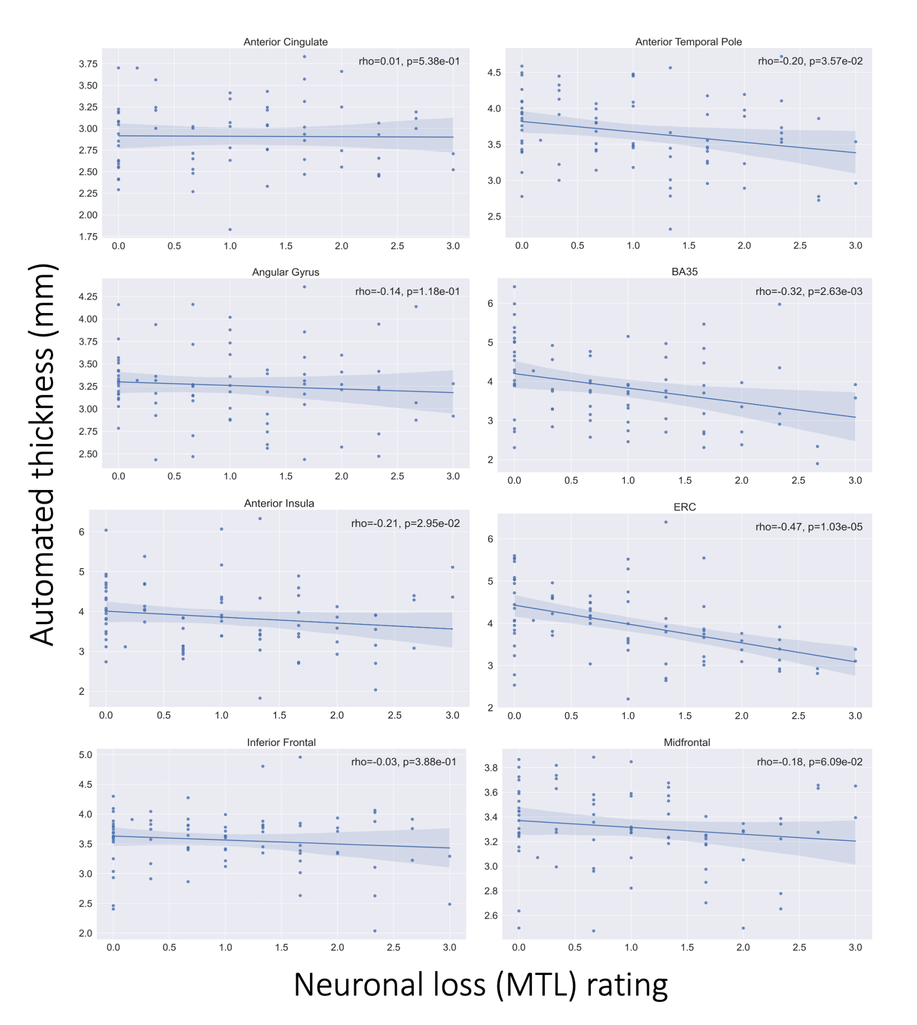

Figure 11: Spearman's correlation between cortical thickness measures derived from topologically corrected nnU-Net-CRUISE gray matter segmentation and medial temporal lobe (MTL) neuronal loss rating with p-value.

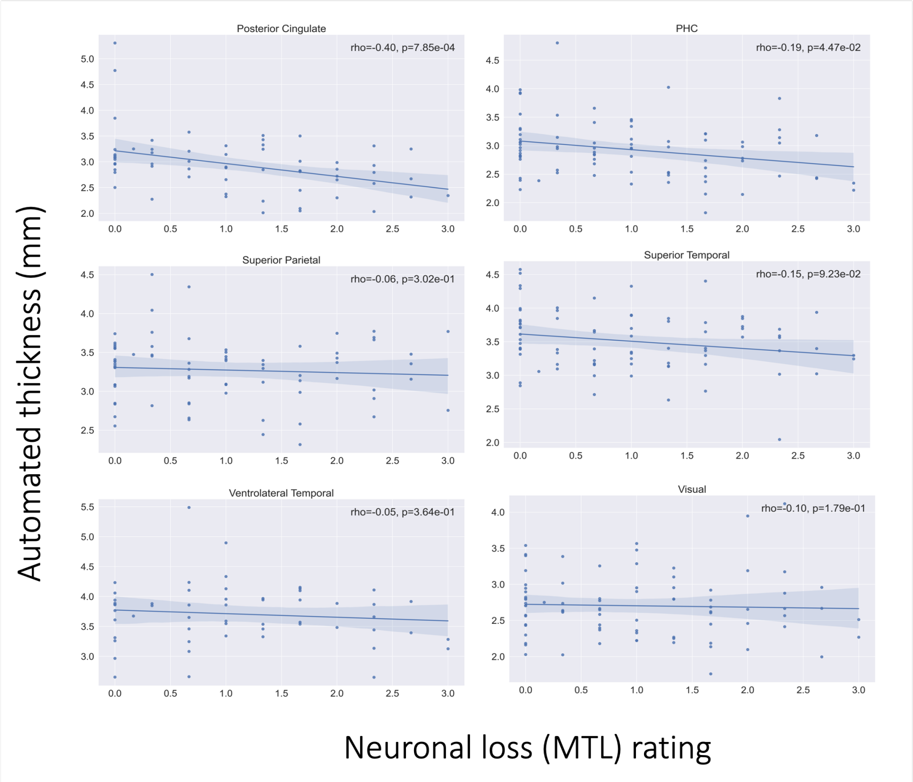

Figure 11: ***Continued.*** Spearman's correlation between cortical thickness measures derived from topologically corrected nnU-Net-CRUISE gray matter segmentation and the medial temporal lobe (MTL) neuronal loss rating with p-value.

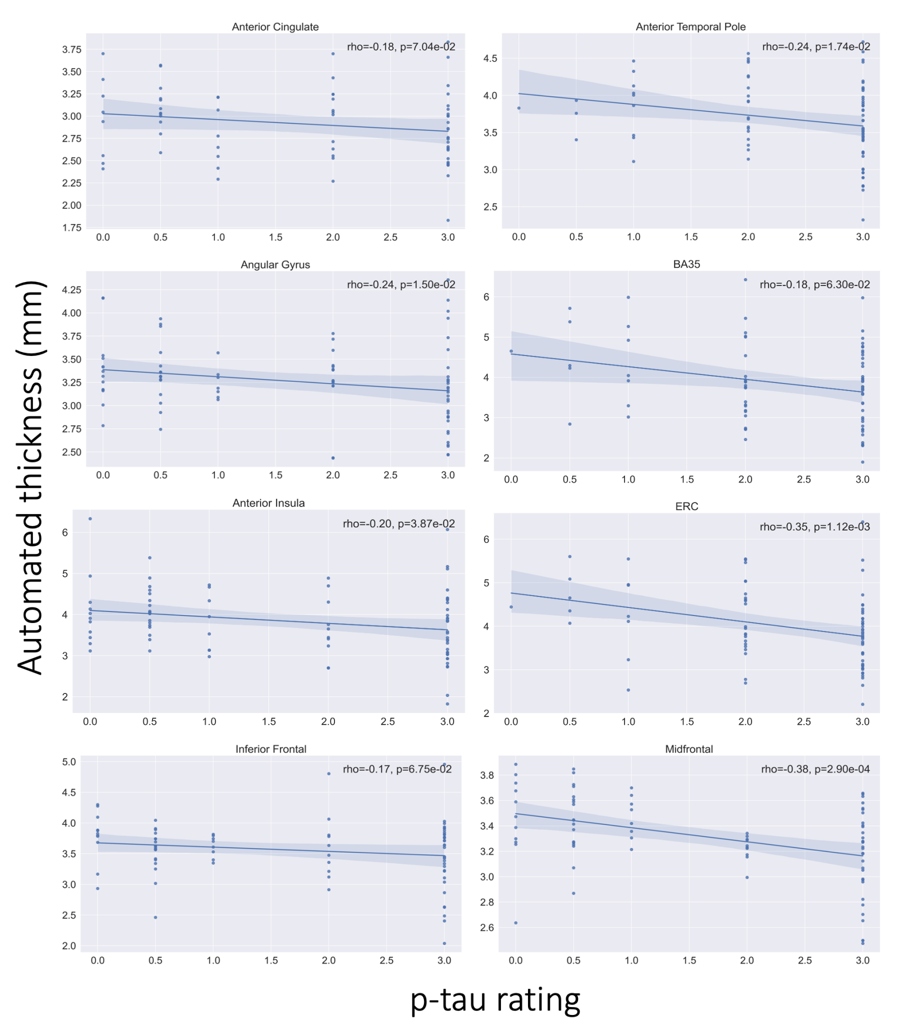

Figure 12: Spearman's correlation between cortical thickness measures derived from topologically corrected nnU-Net-CRUISE gray matter segmentation and regional p-tau rating with p-value.

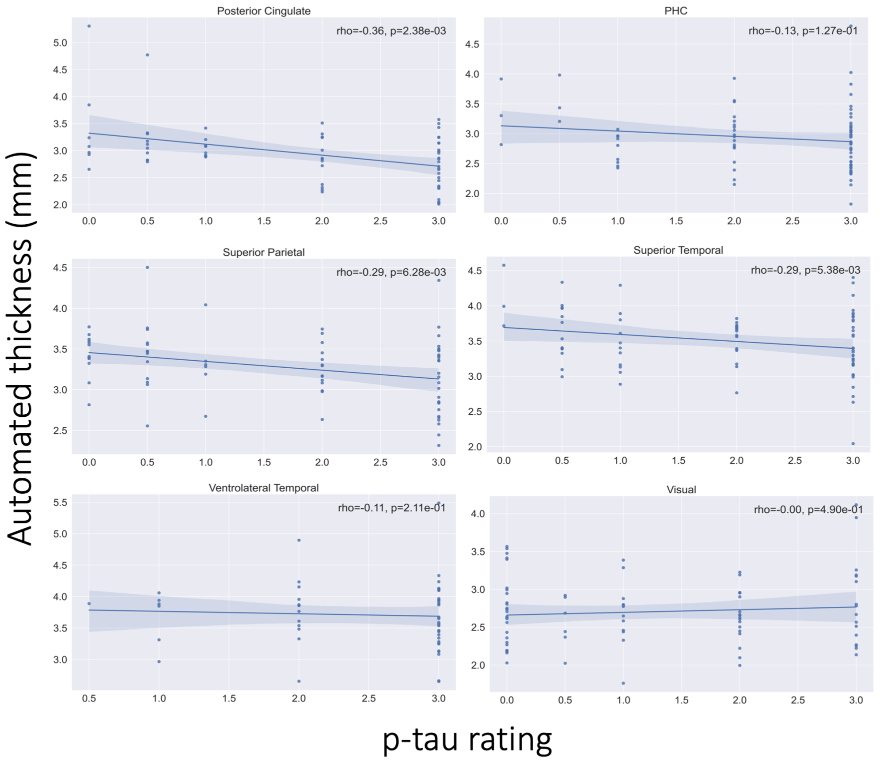

Figure 12: ***Continued.*** Spearman's correlation between cortical thickness measures derived from topologically corrected nnU-Net-CRUISE gray matter segmentation and regional p-tau rating with p-value.

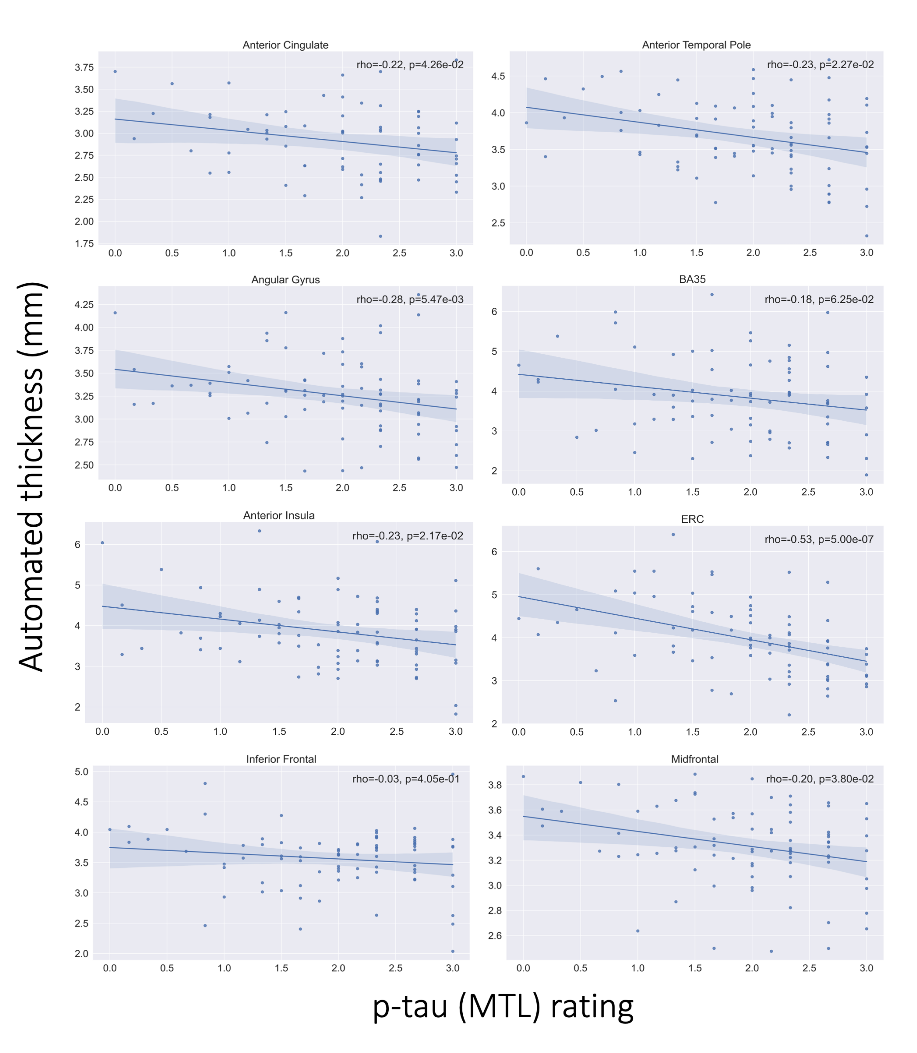

Figure 13: Spearman's correlation between cortical thickness measures derived from topologically corrected nnU-Net-CRUISE gray matter segmentation and medial temporal lobe (MTL) p-tau rating with p-value.

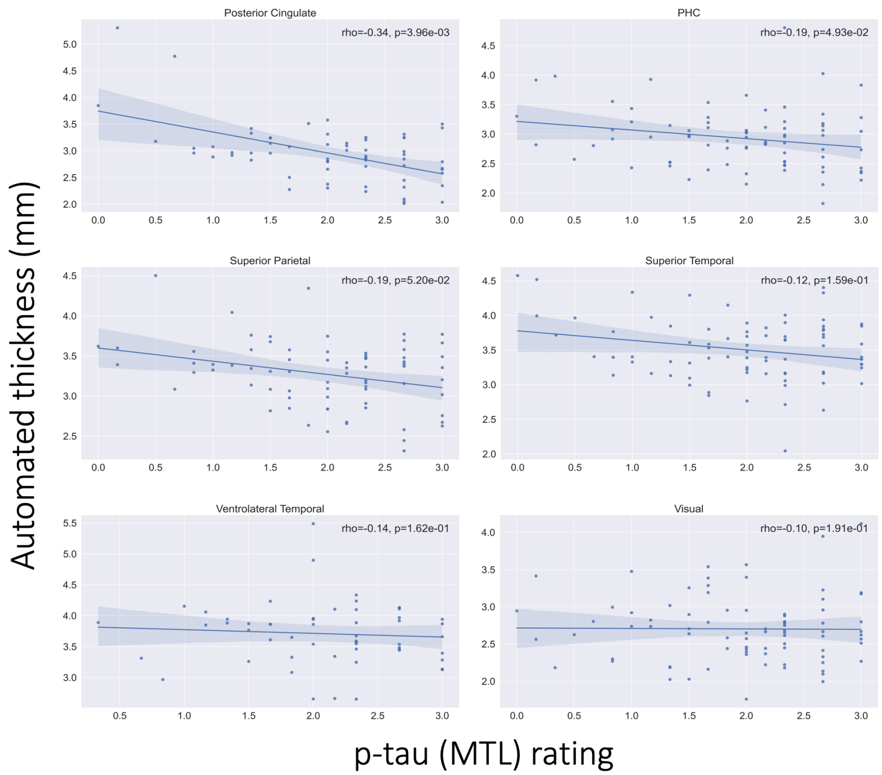

Figure 13: ***Continued.*** Spearman's correlation between cortical thickness measures derived from topologically corrected nnU-Net-CRUISE gray matter segmentation and medial temporal lobe (MTL) p-tau rating with p-value.

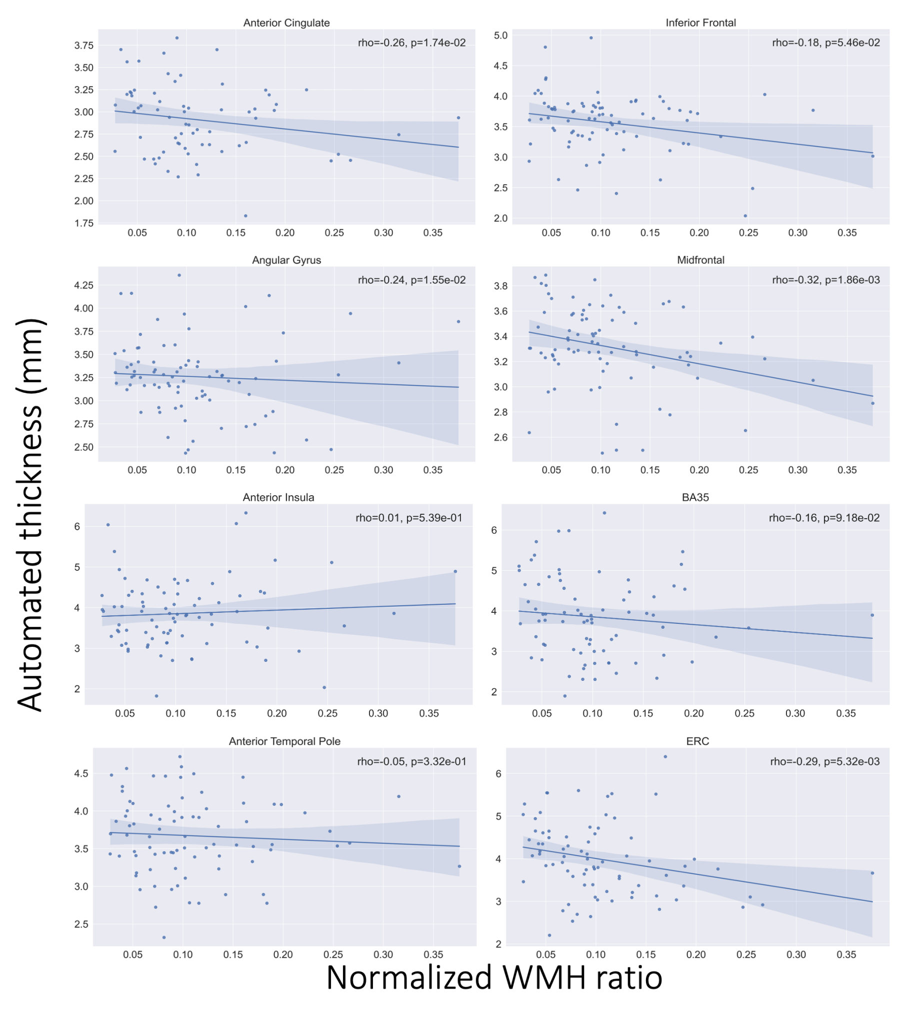

Figure 14: Spearman's correlation between cortical thickness measures derived from topologically corrected nnU-Net-CRUISE gray matter segmentation and the normalized white matter hyperintensities volume.

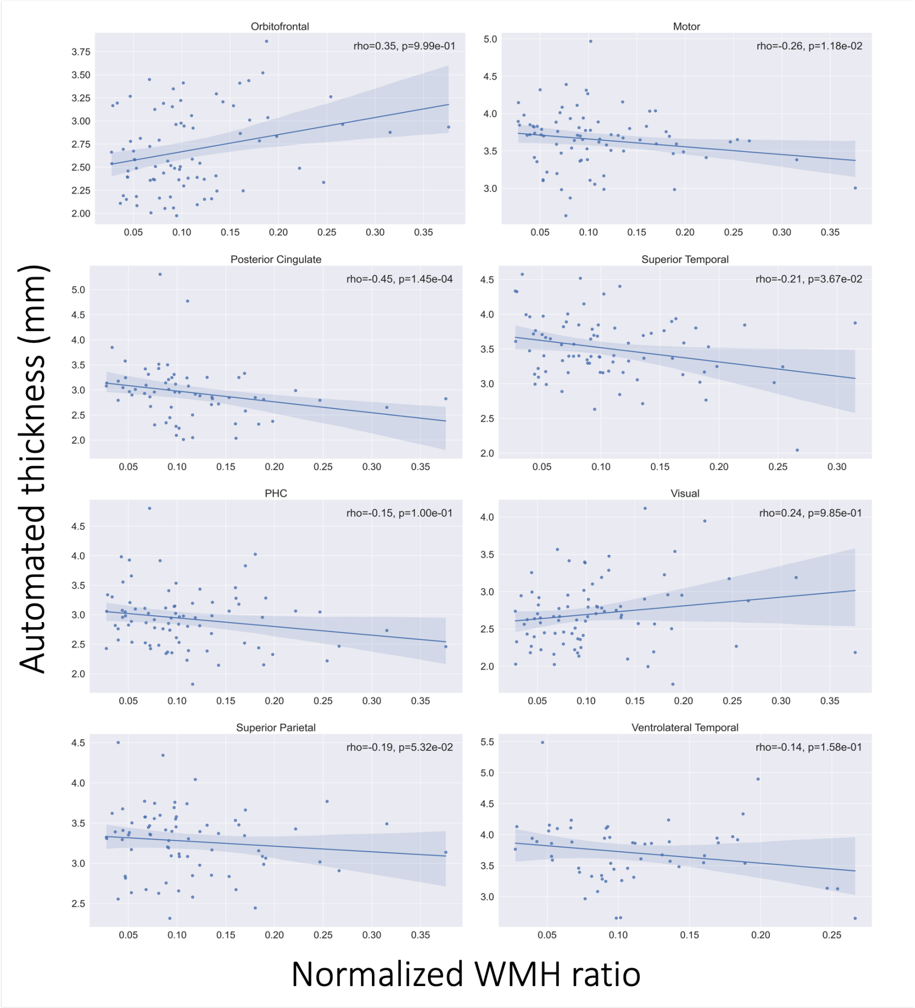

Figure 14: ***Continued.*** Spearman's correlation between cortical thickness measures derived from topologically corrected nnU-Net-CRUISE gray matter segmentation and the normalized white matter hyperintensities volume.

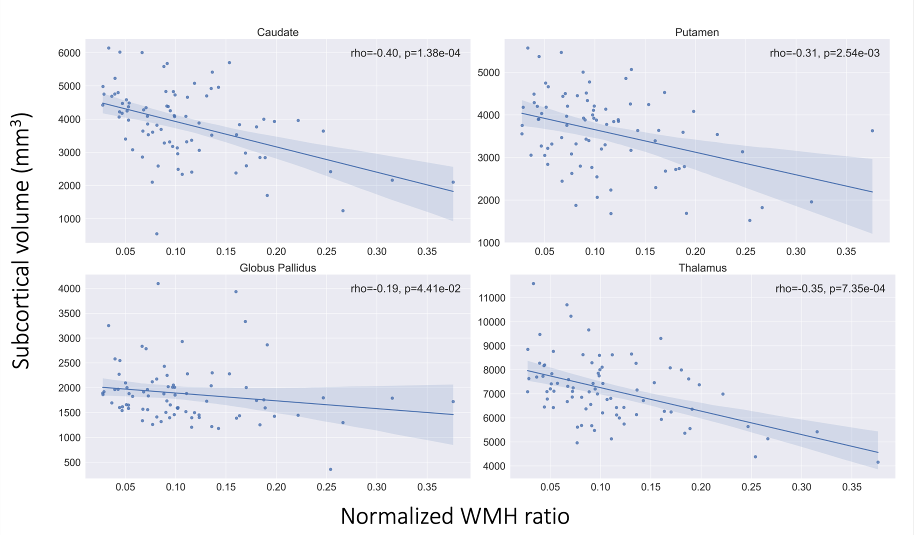

Figure 15: Spearman's correlation between subcortical structure volume and the normalized white matter hyperintensities volume.

References

Bonferroni, C.E., 1935. Il calcolo delle assicurazioni su gruppi di teste. Studi in onore del professore salvatore ortu carboni , 13–60.

Chen, H., Dou, Q., Yu, L., Qin, J., Heng, P.A., 2018. Voxresnet: Deep voxelwise residual networks for brain segmentation from 3d mr images. NeuroImage 170, 446–455.

Dawe, R.J., Bennett, D.A., Schneider, J.A., Vasireddi, S.K., Arfanakis, K., 2009. Postmortem mri of human brain hemispheres: T2 relaxation times during formaldehyde fixation. Magnetic Resonance in Medicine: An Official Journal of the International Society for Magnetic Resonance in Medicine 61, 810–818.

Isensee, F., Jaeger, P.F., Kohl, S.A., Petersen, J., Maier-Hein, K.H., 2021. nnu-net: a self-configuring method for deep learning-based biomedical image segmentation. Nature methods 18, 203–211.

Khandelwal, P., Yushkevich, P., 2020. Domain generalizer: A few-shot meta learning framework for domain generalization in medical imaging, in: Domain Adaptation and Representation Transfer, and Distributed and Collaborative Learning. Springer, pp. 73–84.

Milletari, F., Navab, N., Ahmadi, S.A., 2016. V-net: Fully convolutional neural networks for volumetric medical image segmentation, in: 2016 fourth international conference on 3D vision (3DV), IEEE. pp. 565–571.

Oktay, O., Schlemper, J., Folgoc, L.L., Lee, M., Heinrich, M., Misawa, K., Mori, K., McDonagh, S., Hammerla, N.Y., Kainz, B., et al., 2018. Attention u-net: Learning where to look for the pancreas. arXiv preprint arXiv:1804.03999 .

Rickmann, A.M., Roy, A.G., Sarasua, I., Navab, N., Wachinger, C., 2019. 'project & excite'modules for segmentation of volumetric medical scans, in: International Conference on Medical Image Computing and Computer-Assisted Intervention, Springer. pp. 39–47.

Roy, A.G., Navab, N., Wachinger, C., 2018. Recalibrating fully convolutional networks with spatial and channel "squeeze and excitation" blocks. IEEE transactions on medical imaging 38, 540–549.

Zhu, W., Huang, Y., Zeng, L., Chen, X., Liu, Y., Qian, Z., Du, N., Fan, W., Xie, X., 2019. Anatomynet: deep learning for fast and fully automated whole-volume segmentation of head and neck anatomy. Medical physics 46, 576–589.
